# Design of adaptive structures through energy minimization: extension to tensegrity

**DOI:** 10.1007/s00158-021-02899-y

**Published:** 2021-07-30

**Authors:** Yafeng Wang, Gennaro Senatore

**Affiliations:** Applied Computing and Mechanics Laboratory (IMAC), School of Architecture, Civil and Environmental Engineering (ENAC), Swiss Federal Institute of Technology (EPFL), CH-1015 Lausanne, Switzerland

**Keywords:** Adaptive structures, Tensegrity structures, Integrated structure-control, Active structural control, Structural optimization, Sustainable building design

## Abstract

This paper gives a new formulation to design adaptive structures through total energy optimization (TEO). This methodology enables the design of truss as well as tensegrity configurations that are equipped with linear actuators to counteract the effect of loading through active control. The design criterion is whole-life energy minimization which comprises an embodied part in the material and an operational part for structural adaptation during service. The embodied energy is minimized through simultaneous optimization of element sizing and actuator placement, which is formulated as a mixed-integer nonlinear programming problem. Optimization variables include element cross-sectional areas, actuator positions, element forces, and node displacements. For tensegrity configurations, the actuators are not only employed to counteract the effect of loading but also to apply appropriate prestress which is included in the optimization variables. Actuator commands during service are obtained through minimization of the operational energy that is required to control the state of the structure within required limits, which is formulated as a nonlinear programming problem. Embodied and operational energy minimization problems are nested within a univariate optimization process that minimizes the structure’s whole-life energy (embodied + operational). TEO has been applied to design a roof and a high-rise adaptive tensegrity structure. The adaptive tensegrity solutions are benchmarked with equivalent passive tensegrity as well as adaptive truss solutions, which are also designed through TEO. Results have shown that since cables can be kept in tension through active control, adaptive tensegrity structures require low prestress, which in turn reduces mass, embodied energy, and construction costs compared to passive tensegrity structures. However, while adaptive truss solutions achieve significant mass and energy savings compared to passive solutions, adaptive tensegrity solutions are not efficient configurations in whole-life energy cost terms. Since cable elements must be kept in tension, significant operational energy is required to maintain stable equilibrium for adaptation to loading. Generally, adaptive tensegrity solutions are not as efficient as their equivalent adaptive truss configurations in mass and energy cost terms.

## Introduction

### Previous work

Adaptive structures are equipped with sensors and actuators to actively counteract the effect of external loads. Sensors are employed to monitor the structure response (e.g., stress, displacements). Actuator actions change internal forces and the structural shape to control the response within required limits. Active systems have been investigated to control the structural response under extreme loading events (Utku 2018). Several systems have been considered including active cable tendons for bridges (Rodellar et al. 2002), active bracing and columns (Reinhorn et al. 1992; Weidner et al. 2018) for buildings, as well as semi-active variable stiffness and damping structural joints (Wang et al. 2020a; b). Active shape control has also been investigated for deployable structures, space cranes, and antennas (Tibert 2002; Veuve et al. 2015).

Tensegrity structures are pin-jointed systems consisting of cables and struts that require appropriate prestress to maintain a stable equilibrium. Generally, tensegrity structures have a high stiffness-to-weight ratio and thus have been investigated as lightweight civil structures such as roof systems (Gilewski 2015), cable domes (Kmet and Mojdis 2015), and pedestrian bridges (Ali et al. 2010). Most optimization methods that have been proposed to design passive tensegrity structures (i.e., not equipped with an actuation system) aim to obtain minimum mass and maximum stiffness solutions. Masic and Skelton (Masic and Skelton 2004) presented an optimization method based on nonlinear programming to obtain tensegrity structures that have an optimal mass-to-stiffness ratio. Chen and Skelton (Chen and Skelton 2020) proposed a general approach based on nonlinear programming to design minimum mass tensegrity structures subject to equilibrium, stress, and buckling constraints. Skelton et al. (Skelton et al. 2014) presented a design methodology to obtain tensegrity bridges that comprise self-similar repetitions of a basic unit inspired by Michell minimum mass solutions for a centrally loaded beam (Michell 1904). Topology optimization methods have also been proposed to design passive tensegrity structures (Kanno 2013; Wang et al. 2020a; b). For example, least-weight tensegrity structures have been obtained through discrete structural topology optimization based on mixed-integer linear programming subject to equilibrium and stress constraints in (Kanno 2013) as well as to buckling constraints in (Xu et al. 2018).

Since cables can only carry tension, appropriate prestress must be applied to maintain stable equilibrium and to ensure that the cable elements do not slack under external loading. Depending on the structure geometry and loading, typically, a high prestress level is required which might be impractical during construction resulting in an increase of labor costs as well as an increase of element cross-sectional areas to ensure that stress and stability limits are met (Quagliaroli et al. 2015). However, if some of the cables or struts in a tensegrity structure are equipped with linear actuators, it is generally possible to control the response under loading without the need of a large prestress because internal forces and node displacements can be modified through active control (Fest et al. 2003). In addition, prestress can be applied directly through controlled length changes of linear actuators that are installed on the structure elements (Adam and Smith 2008).

Integrated structure-control optimization methods for tensegrity systems have been formulated with the objective to minimize control energy, structure mass, or a combination of both. Masic et al. (Masic et al. 2005) presented a method for prestress optimization of tensegrity structures to obtain an optimal linear-quadratic regulator (LQR) performance. Raja and Narayanan (Raja and Narayanan 2007) employed optimal control theory based on H_2_ and H_∞_ controller with full-state and limited-state feedback for vibration suppression of tensegrity structures equipped with piezoelectric actuators. In (Raja and Narayanan 2009), the control method given in (Raja and Narayanan 2007) was nested within an optimization strategy based on a genetic algorithm to improve the controller performance. Li et al. (Li et al. 2011) formulated a method for class 1 and class 2 tensegrity systems to minimize simultaneously mass and control energy, given a predetermined set of locations for sensors and actuators as well as constraints on the system response. The design of a pedestrian bridge made of six ring-shaped tensegrity modules and spanning 21.6 m was presented in (Ali et al. 2010). Each module comprised 15 struts held together by 30 cables. Cable prestress and element cross-sectional areas were treated as design variables. A genetic algorithm was employed to minimize a cost function of the material member monetary cost multiplied by a factor to account for the effect of prestress on construction cost. Design constraints included stress and element buckling limits as well as deflection limits. A minimum cost solution was obtained that satisfied all constraints. It was found that including prestress in the design variables is important because minimum material cost solutions generally require a high level of prestress, which usually increases construction costs.

In most existing design methods for adaptive tensegrity structures, the actuator positions (i.e., actuator placement or layout) have been set a priori to avoid the need for a mixed-integer problem formulation. Alternatively, sequential approaches have been proposed to optimize structure layout and actuator placement separately. These methods therefore cannot guarantee solution optimality which involves obtaining structure and actuator layouts simultaneously as a solution of a mixed-integer problem.

Generally, well-designed adaptive structures are able to operate closer to capacity owing to the ability to reduce the effect of loading through active control instead of solely through passive load-bearing resistance (i.e., material and form) (Teuffel 2004; Sobek 2016). This way, through adaptation, structures can be designed with a significantly better material utilization compared to passive structures (Böhm et al. 2019; Reksowardojo et al. 2020). However, adaptive structures might require significant energy for adaptation to loading during service. Hence, material savings might come at a high total energy cost. To address this challenge, Senatore et al. (Senatore et al. 2019) proposed an integrated structure-control optimization method to design adaptive pin-jointed structures through “whole-life” energy minimization. Whole-life energy (i.e., total energy) consists of an embodied part in the material and an operational part for structural adaptation during service. Numerical and experimental testing has shown that minimum energy adaptive structures not only have a better material utilization but also a much lower energy impact and thus a lower overall environmental impact. This methodology was applied to design spatial structures of complex layout, showing that minimum energy adaptive structures can save up to 40–50% of the total energy for stiffness governed designs (e.g., long-span bridges and high-rise structures) compared to weight-optimized passive structures (Senatore et al. 2018a; Senatore and Reksowardojo 2020). Experimental testing confirmed numerical predictions (Senatore et al. 2018b; Reksowardojo et al. 2019) showing that the ability to reduce deflections actively through shape control enables new designs such as super slender high-rise structures. In (Senatore et al. 2019), whole-life energy minimization was decoupled into embodied and operational energy minimization. Embodied energy minimization was further decoupled into two subproblems which are structural sizing and actuator placement optimization. For this reason, solution optimality could not be guaranteed. Wang and Senatore (Wang and Senatore 2020a) presented a reformulation of the method given in (Senatore et al. 2019) whereby all design variables (i.e., cross-sectional areas, actuator positions, and control commands) are optimized simultaneously through an all-in-one model based on mixed-integer nonlinear programming (MINLP). A comparison of the two approaches was carried out in (Wang and Senatore 2020a) showing that both methods produce solutions that are only marginally different in energy cost terms. However, the nested formulation is significantly more efficient in computation time terms, which allows obtaining solutions for structures of complex layout within a fraction of the time required by the all-in-one formulation.

The methods given in (Senatore et al. 2019; Wang and Senatore 2020a) have been formulated for pin-jointed configurations in which all elements can take tension and compression, and thus there is no need to include prestress to ensure cable elements do not slack during adaptation to loading (i.e., stable equilibrium). For this reason, these methods cannot be directly applied to design adaptive tensegrity structures through energy minimization.

### New contribution

The design of adaptive tensegrity structures through energy minimization has received little attention. In none of the existing methods, the total energy (embodied + operational) required by the structure for the entire service life has been explicitly set as the objective function. No rigorous study has been carried out to benchmark energy and material costs of an active tensegrity structure with those of equivalent passive designs.

The work described in this paper provides an answer to these open research gaps. A new general method is formulated to design minimum energy adaptive structures including tensegrity configurations. Prestress is added to the optimization variables which include element sizing, actuator positions as well as controlled and uncontrolled states (forces and displacements) under loading. The actuators are not only employed to counteract the effect of loading but also to apply appropriate prestress to ensure self-equilibrium, which enables the design of adaptive tensegrity structures. This new method is applied to design a roof and a high-rise adaptive tensegrity structure, which are benchmarked in mass and energy cost terms against weight-optimized passive tensegrity and truss structures.

### Outline

The paper is organized as follows. Section 2 outlines a methodology to design adaptive tensegrity structures through energy minimization. Section 3 gives a formulation to apply prestress through controlled length changes of linear actuators that are installed in series with the structural elements. Sections 4 and 5 give embodied and operational energy minimization formulations, respectively. Section 6 gives the whole-life energy minimization formulation which coordinates embodied and operational energy minimization problems. Section 7 presents applications of the design methodology through numerical examples. Sections 8 and 9 conclude this paper.

## Synthesis of minimum energy adaptive structures

### Total energy minimization (TEO)

This work builds on the formulations given in (Senatore et al. 2019; Wang and Senatore 2020a) by adopting the whole-life energy criterion to design adaptive structures. As for (Senatore et al. 2019), whole-life energy minimization is decoupled into two subproblems: embodied and operational energy minimization. Since it was proven numerically that the all-in-one formulation given in (Wang and Senatore 2020a) and the nested formulation given in (Senatore et al. 2019) produce similar solutions in energy cost terms, a sequential approach is adopted in this work to reduce optimization complexity. The all-in-one formulation (Wang and Senatore 2020a) involves a very large number of variables as well as strong nonlinearity, and therefore, it can only be applied to relatively small-sized design problems. However, different from the nested formulation given in (Senatore et al. 2019), in this work element sizing and actuator placement are optimized simultaneously. This way, the actuator system embodied energy (and thus the mass) is directly included in the embodied energy minimization process which is formulated as a mixed-integer programming problem. Element cross-sectional areas and actuator positions are the primary design variables while internal forces and node displacements are treated as state variables (Section 4). For tensegrity configurations, prestress is included in the optimization variables, and it is applied directly through actuation. Different from (Senatore et al. 2019; Wang and Senatore 2020a), operational energy minimization is formulated as a nonlinear programming problem. The objective function is the minimization of the actuator work that is required for structural adaptation during service. The optimization variables are the actuator commands to control the response of the structure within required stress, stability, and deflection limits (Section 5).

**Fig. 1:**
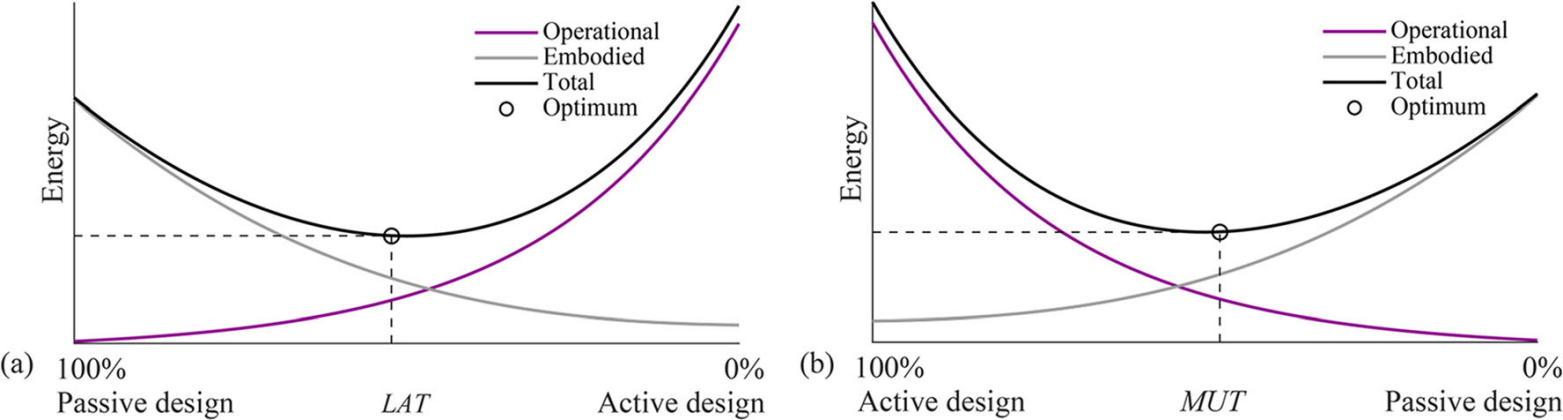
Embodied, operational, and total energy as a function of load activation threshold (*LAT*)

In (Senatore et al. 2019), embodied and operational energy minimization is coordinated through an auxiliary variable denoted as material utilization (*MUT*). The *MUT* is the demand-to-capacity ratio for the structure as a whole, which is defined in the range 0% < *MUT* ≤ 100% in percentage terms with respect to the admissible stress. *MUT* is related to another auxiliary variable called the load activation threshold (*LAT*). *LAT* was defined in (Senatore et al. 2019) as the lowest intensity loading event that causes a violation of stress and/or displacement limits. *LAT* is defined in the range 0% ≤ *LAT* ≤ 100% in percentage terms with respect to the maximum expected load during service. Figure 1a and b show a generic plot of whole-life, embodied, and operational energy as functions of *LAT* and MUT, respectively.

**Fig. 2:**
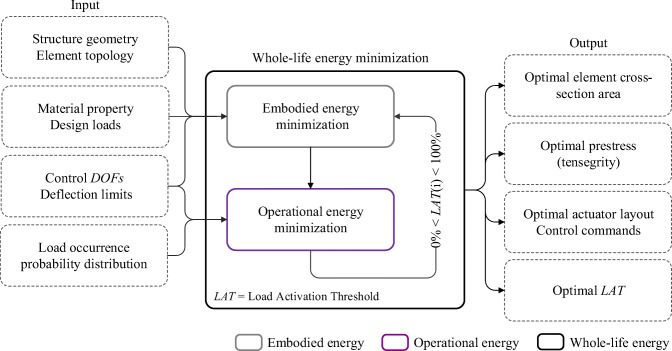
Design of minimum-energy adaptive structures

Structural adaptation is required to counteract the effect of loading events that are larger than *LAT*. *MUT* and *LAT* are in a one-to-one correspondence. Low embodied energy structures (lightweight and flexible, high *MUT*) are usually characterized by a low *LAT* because active control is required to counteract low-intensity loading events which are likely to occur relatively often. On the contrary, high embodied energy structures (high mass, stiff, low *MUT*) are characterized by a high *LAT.* While in (Senatore et al. 2019), *LAT* was treated as a state variable, in this work, embodied and operational energy minimization is nested within a univariate optimization process whose variable is the *LAT* (Section 6). *LAT* is varied in a predefined range. For each *LAT*, a new configuration is obtained by minimizing embodied and operational energy. The configuration of minimum total energy (embodied + operational) is then selected as the optimum solution. The formulation given in this work allows to explicitly assign *LAT* according to practical design requirements. For example, *LAT* could be chosen based on the expected frequency of occurrence of a certain loading event as well as the expected life cycle of control system components. This methodology can be applied to obtain minimum energy truss as well as tensegrity configurations. For tensegrity configurations, cable and strut topology must be defined as part of the input element topology, and the output includes the optimal prestress state. The total energy optimization (TEO) process is illustrated in the flowchart shown in Fig. 2.

### Loading conditions

**Fig. 3:**
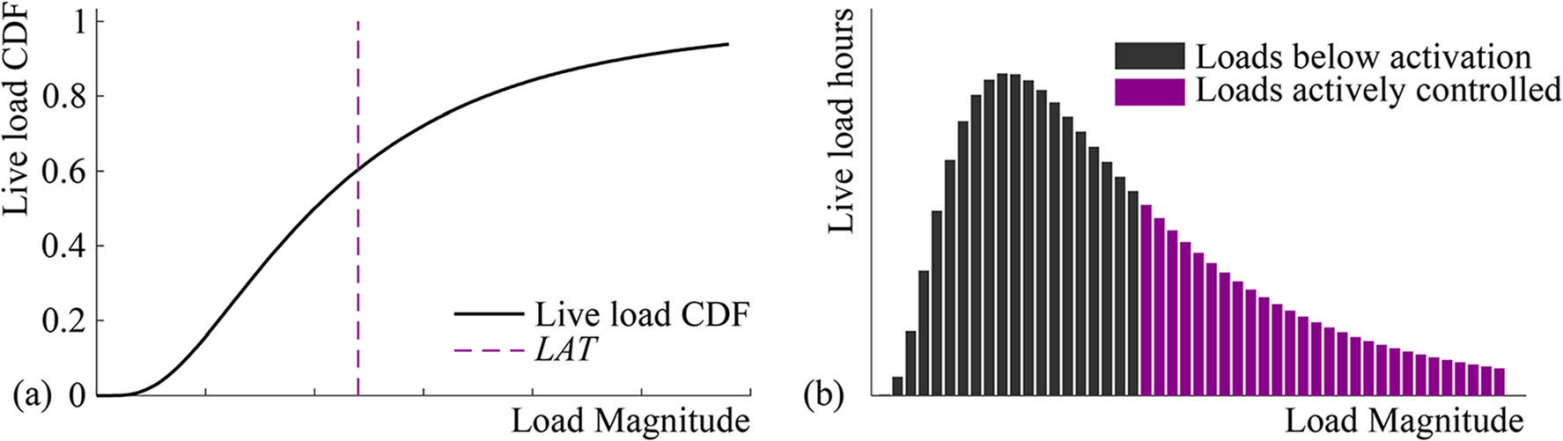
**a** Live load cumulative distribution function (CDF); **b** load event duration (Senatore et al. 2019)

To compute the operational energy that is required for structural adaptation during service, a probability occurrence of the load must be defined. The structure is assumed to be subjected to a permanent **P**^*permanent*^and a randomly fluctuating live load **P**^*live*^. For simplicity, all loads that are not permanent are considered as live loads including events such as high winds and unusual crowds. Following (Senatore et al. 2019), the live load probability distribution is modeled with a log-normal function which is suitable for a generic random loading occurrence. For specific loading events, other probability distributions should be adopted. Figure 3a shows the plot of a generic log-normal cumulative distribution. Assume there are *n*^*p*^ load cases and *j* indicates the *j*^th^ load case. The load probability distribution is discretized into *n*^*d*^ bins. The load event corresponding to the *k*^*th*^ bin (i.e., occurrence) is denoted as ${\mathbf{P}}_{jk}^{live}$. The characteristic value is set based on the strongest intensity loading event ${\mathbf{P}}_{jd}^{live}$(i.e., design load) that is expected during service. To define the log-normal probability distribution, the characteristic value is typically set to the 95th percentile of the associated normal distribution. However, the characteristic value could be varied depending on the expected probability of occurrence of each load case. For simplicity, the mean *μ* of the associated normal distribution is set to zero. Once the mean and the characteristic value are set, the standard deviation (*SD*) of the associated normal distribution can be computed as

1 $$ S{D}_j=\frac{\log \left(\max \left({\mathbf{P}}_{jd}^{live}\right)\right)-\mu }{\Phi^{-1}(0.95)} $$

where Φ^−1^is the inverse of the cumulative distribution function of a standard normal distribution. *LAT* is indicated by a dashed line in Fig. 3a. As discussed in 2.1, *LAT* is the lowest intensity loading occurrence that causes a violation of stress and/or displacement limits. *LAT* is defined in percentage terms with respect to the design load ${\mathbf{P}}_{LAT}^{live}= LAT\cdot {\mathbf{P}}_d^{live}$. Figure 3b shows the plot of the discretized probability density scaled by the expected service life which is typically set to 50 years. The duration of each loading event Δ*t*_*jk*_ is obtained through scaling the expected structure service life with the *k*^th^ occurrence probability for the *j*^th^ load case.

### Structural adaptation phases

**Fig. 4:**
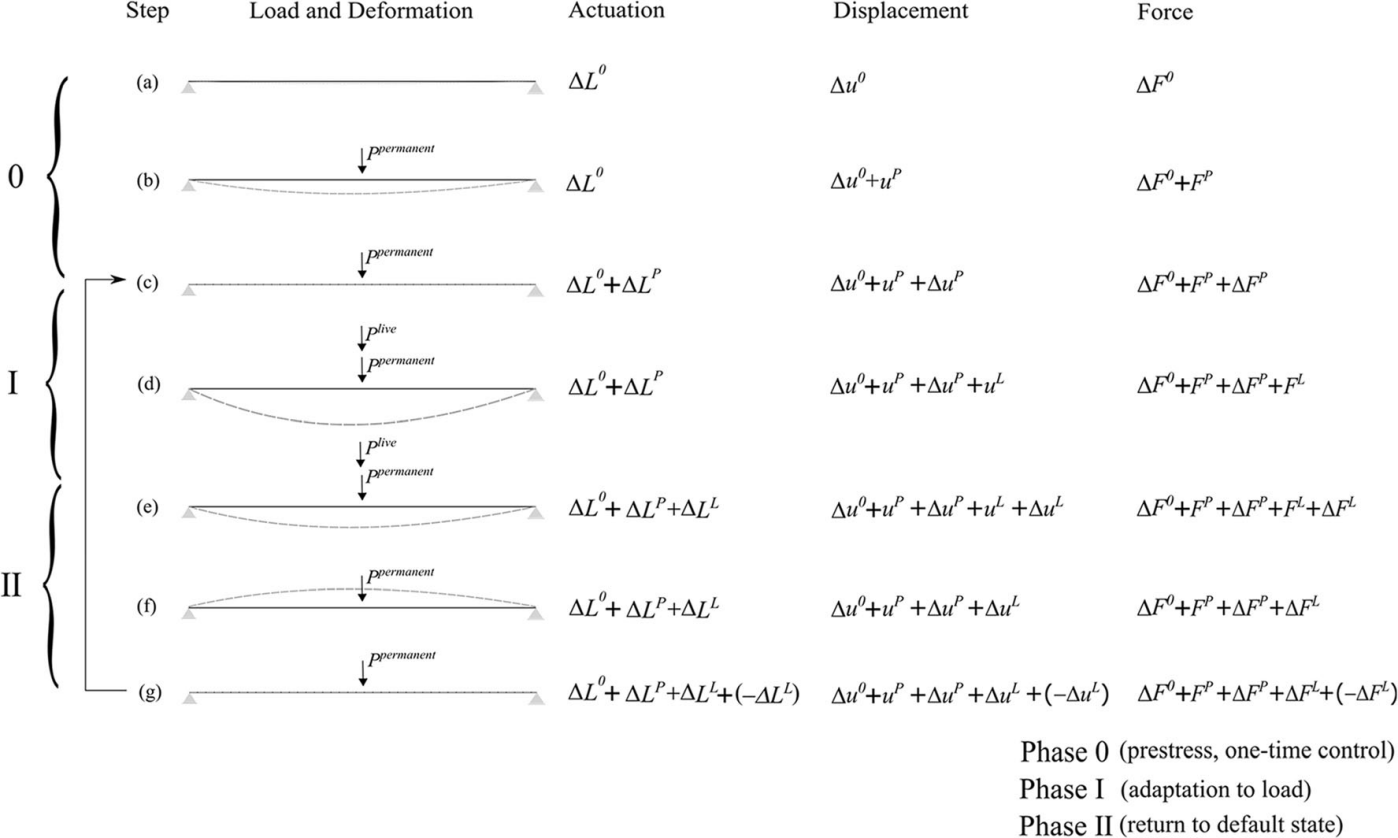
Structural adaptation process

The actuation system comprises linear actuators. A linear actuator is installed in series with a structural element. The actuators modify internal forces and node displacements through length changes. Since an actuator is installed in series, the element force and the actuator force are the same. In other words, the actuator changes length while subjected to a force that is identical to that of the element onto which it is installed. Figure 4 shows a conceptual diagram of the structural adaptation process. For simplicity, the structure is indicated by a single line, and the actuation system is not represented. There are three phases: phase 0, phase I, and phase II.

Phase 0 adaptation is performed to apply prestress (for tensegrity configurations) and counteract the effect of **P**^*permanent*^. Forces and displacements at phase 0 completion are the default state (state c). In step (a), the structure is prestressed through a first actuator length change **Δ*L***^*0*^ that causes a change of forces **Δ*F***^*0*^ to ensure self-equilibrium before external loading is applied. In step (b), actuator command **Δ*L***^*P*^ is applied to control element forces and node displacements within required limits under permanent load. Phase 0 step (b) can be thought of as a pre-cambering process to ensure that deflections under **P**^*permanent*^ are kept as small as possible. Note that phase 0 adaptation is performed only once during construction and before service. Phase I adaptation (c–e) is performed through **Δ*L***^*L*^ to counteract the effect of **P**^*live*^. Phase II adaptation (e–g) is performed through -**Δ*L***^*L*^ to eliminate residual effects caused by actuation in phase 1 after **P**^*live*^ is removed so that the structure can return to the default state (state g is identical to state c). Since element forces, node displacements and actuator length changes are obtained from the superposition of different terms, it is convenient to refer to each state through superscript *ψ* as

2 $$ {\displaystyle \begin{array}{c}{\mathbf{F}}^{\psi },\forall \psi \in \left\{a,b,c,d,e,f,g\right\}\\ {}{\mathbf{u}}^{\psi },\forall \psi \in \left\{a,b,c,d,e,f,g\right\}\\ {}\boldsymbol{\Delta} {\mathbf{L}}^{\psi },\forall \psi \in \left\{a,b,c,d,e,f,g\right\}\end{array}}. $$

### Model assumptions

The following assumptions are adopted:

- Following (Skelton and Oliveira 2009), a general definition of tensegrity structures is adopted in this study. That is, a tensegrity structure is a pin-jointed system consisting of cables and struts which require appropriate prestress to maintain stable equilibrium under loading, i.e., cable elements do not slack. Only kinematically determinate tensegrity systems are considered in this work, i.e., which do not contain mechanisms.
- Elements are pin-jointed and all loads are transferred to nodes as point loads.
- The formulation is implemented with the assumption of small strains and small displacements. To prevent potential stability issues caused by finite mechanisms that could develop through cable slackness, cable elements are kept in tension under all loading events through appropriate prestress and control actions.
- The structure dynamic response is assumed to be controlled by other means; hence, seismic design criteria are not considered. In addition, fatigue is not considered as a limit state because the structure is designed to be controlled under strong loading events which occur rarely.

## Prestress applied through actuation

Consider a tensegrity structure that comprises *n*^*e*^ elements and *n*^*n*^ joints in *d*-dimensions. The number of free degrees of freedom is *n*^*f*^ = *dn*^*n*^ - *n*^*c*^ where *n*^*c*^ is the number of support conditions. Prestress must be applied in a tensegrity system to maintain self-equilibrium by ensuring tension and compression states in cables and struts, respectively (Skelton and Oliveira 2009; Zhang and Makoto 2015). Denote ${\mathbf{F}}_0\in {\mathrm{\mathbb{R}}}^{n^e\times 1}$ the prestress state before external loads are applied, then self-equilibrium conditions are

3 $$ \mathbf{A}{\mathbf{F}}_0=0, $$

where $\mathbf{A}\in {\mathrm{\mathbb{R}}}^{n^f\times {n}^e}$ is the structure equilibrium matrix which contains all element direction cosines (Pellegrino and Calladine 1986). Denote *r* the rank of **A**, then the number of self-stress states is *s* = *n*^*e*^ – *r*. From (3), it is clear that **F**_0_ must lie in the null space of **A**; thus, **F**_0_ can be expressed as a linear combination of the self-stress states

4 $$ {\mathbf{F}}_0={\mathbf{W}}_s\boldsymbol{\upbeta}, $$

where ${\mathbf{W}}_s\in {\mathrm{\mathbb{R}}}^{n^e\times s}$ contains the *s* self-stress states column-wise (**W**_*s*_ is the basis of the null space of **A**) (Pellegrino and Calladine 1986) and **β** ∈ ℝ^*s* × 1^ is a combination coefficient vector. In existing prestress optimization methods for tensegrity structures (Xu and Luo 2010; Wang and Senatore 2020b), the combination coefficient vector **β** is usually treated as the primary design variable. A suitable combination coefficient **β** is obtained to satisfy the unilaterality condition on element forces (struts in compression and cables in tension). However, (4) can only produce a theoretical solution because structural parameters such as cross-sectional areas, material properties, and element lengths are not considered. In practice, geometric compatibility and imperfections (e.g., lack of fit) must be considered to apply the required prestress.

Since this work is concerned with the design of adaptive tensegrity structures that are equipped with linear actuators, it is convenient to apply prestress directly through actuation. Assume a given actuator layout; i.e., a certain number of linear actuators are installed in series with selected elements. The action of a linear actuator is a length change that, generally, modifies internal forces $\Delta \mathbf{F}\in {\mathrm{\mathbb{R}}}^{n^e\times 1}$ and node displacements $\Delta \mathbf{u}\in {\mathrm{\mathbb{R}}}^{n^{f\times 1}}$. The actuator length change is modeled as an inelastic change of length $\varDelta \mathbf{L}\in {\mathrm{\mathbb{R}}}^{n^e\times 1}$ of the element onto which it is installed. *Δ***L** is added to the element elastic deformation caused by the change of force Δ**F** such that the total element deformation ${\mathbf{e}}^t\in {\mathrm{\mathbb{R}}}^{n^e\times 1}$ is

5 $$ {\mathbf{e}}^t=\mathbf{e}+\Delta \mathbf{L} $$

where **e** = **G**Δ**F** is the elastic deformation and $\mathbf{G}\in {\mathrm{\mathbb{R}}}^{n^f\times {n}^f}$ is the flexibility matrix. The flexibility matrix is diagonal for pin-jointed structures with entries *L*_*i*_/*E*_*i*_*α*_*i*_; *L*_*i*_,*E*_*i*_, and *α*_*i*_ are length, Young modulus, and cross-sectional area of the *i*^th^ element, respectively. The compatibility condition (Pellegrino and Calladine 1993) given by the orthogonality between self-stress states **W**_*s*_ and the element total deformation **e**^*t*^ is

6 $$ {\mathbf{W}}_s^{\mathrm{T}}\left(\mathbf{e}+\Delta \mathbf{L}\right)=0 $$

Note that (6) holds for structures whose rigid body motion is constrained by supports as well as free-standing structures (Senatore and Reksowardojo 2020). Replacing (4) into (6) and solving for **β** gives

7 $$ \boldsymbol{\upbeta} =-{\left({\mathbf{W}}_s^{\mathrm{T}}\mathbf{G}{\mathbf{W}}_{\mathbf{s}}\right)}^{-1}{\mathbf{W}}_s^{\mathrm{T}}\Delta \mathbf{L} $$

Therefore, Δ**F** can be computed as

8 $$ \Delta \mathbf{F}=-{\mathbf{W}}_s{\left({\mathbf{W}}_s^{\mathrm{T}}\mathbf{G}{\mathbf{W}}_s\right)}^{-1}{\mathbf{W}}_s^{\mathrm{T}}\boldsymbol{\Delta} \mathbf{L}. $$

Equation (8) proves that the actuator length change Δ**L** can be employed directly to apply the required prestress **F**_0_. Generally, (8) relates the actuator command Δ**L** to the change of element forces Δ**F** that it causes.

An alternative formulation can be derived from force equilibrium conditions (9) and compatibility between node displacements and element deformation (10)

9 $$ \mathbf{A}\Delta \mathbf{F}=\mathbf{0} $$

10 $$ {\mathbf{A}}^{\mathrm{T}}\Delta \mathbf{u}=\mathbf{e}+\Delta \mathbf{L} $$

From constitutive relations, Δ**F** can be also computed as

11 $$ \Delta \mathbf{F}=\overline{\mathbf{K}}{\mathbf{L}}^{-1}\mathbf{e} $$

where $\overline{\mathbf{K}}\in {\mathrm{\mathbb{R}}}^{n^f\times {n}^f}$ and $\mathbf{L}\in {\mathrm{\mathbb{R}}}^{n^e\times 1}$ are diagonal matrices whose *i*^th^ diagonal entry is *E*_*i*_*α*_*i*_ and *L*_*i*_, respectively. Then replacing (10) into (11) gives

12 $$ \varDelta \mathbf{F}=\overline{\mathbf{K}}{\mathbf{L}}^{-1}\left({\mathbf{A}}^{\mathrm{T}}\Delta \mathbf{u}-\Delta \mathbf{L}\right) $$

Equation (12) is equivalent to (8) and relates Δ**L** with the change of element forces Δ**F** and node displacements Δ**u** that it causes. However, (12) reduces nonlinearity compared to (8) which is a reciprocal function (i.e., $\mathbf{G}={\overline{\mathbf{K}}}^{-1}\mathbf{L}$) of the element cross-sectional area **α**. The formulation given here to apply prestress through actuation will be integrated into the embodied energy minimization model including other requirements such as unilaterality condition on cable element forces as well as stress and deflection limits under external loading. Therefore, the adoption of (12) instead of (8) helps to reduce optimization complexity.

## Embodied energy minimization

### Objective function

The embodied energy is minimized through a simultaneous optimization of element sizing and actuator positions. Suppose the actuator system is made of *n*^*a*^ linear actuators. The objective function comprises two parts: the energy embodied in *n*^*e*^ structure elements and the energy embodied in *n*^*a*^ actuators. The structure embodied energy is

13 $$ {E}_{emb}^s=\sum \limits_{i=1}^{n^e}{\alpha}_i{L}_i{\rho}_i{e}_i^e, $$

where *α*_*i*_, *L*_*i*_, *ρ*_*i*_, and ${e}_i^e$ are the cross-sectional area, length, material density, and energy intensity factor of the *i*^th^ element, respectively. In (13), the element cross-sectional areas **α** are optimization variables, and all other terms are constant. The energy intensity factor ${e}_i^e$ (MJ/kg) is the energy per unit mass for extraction and manufacturing (Hammond and Jones 2008).

Following (Senatore et al. 2019), it is assumed that the mass of an actuator is a linear function of the force capacity, i.e., the maximum force required for control, with a proportional constant *c* = 0.1 kg/kN (ENERPAC 2016). Therefore, the actuation system embodied energy is

14 $$ {E}_{emb}^a=c\sum \limits_{i=1}^{n^e}{\tilde{F}}_i{n}_i{e}_i^a, $$

where ${\tilde{F}}_i$ and ${e}_i^a$ are force capacity and energy intensity factor of the *i*^th^ actuator; *n*_*i*_ is the *i*^th^ entry of $\mathbf{n}\in {\left\{0,1\right\}}^{n^e\times 1}$ which is a vector of binary variables for the actuator positions. For clarity, when *n*_*i*_ = 1, the *i*^th^ element is equipped with an actuator. Since *n*_*i*_ = 0 for elements that are not equipped with an actuator, the product of vectors $\tilde{F}\in {\mathrm{\mathbb{R}}}^{n^e\times 1}$ by $\mathbf{n}\in {\left\{0,1\right\}}^{n^e\times 1}$ returns the force capacity of all actuators installed in the structure. Actuator force capacity $\tilde{F}$ and actuator positions **n** are optimization variables. Note that the summation is over all elements *n*^*e*^ instead of the total number of actuators *n*^*a*^ because the actuator positions are variables. The total structure embodied energy is given by

15 $$ {E}_{emb}={E}_{emb}^s+{E}_{emb}^a. $$

### Equilibrium and compatibility constraints

Force equilibrium and geometric compatibility constraints for the prestress state, which are expressed by (9) and (12), are here grouped together

16 $$ \Big\{{\displaystyle \begin{array}{c}\mathbf{A}\Delta {\mathbf{F}}^0=\mathbf{0}\\ {}\Delta {\mathbf{F}}^0=\overline{\mathbf{K}}{\mathbf{L}}^{-1}\left({\mathbf{A}}^{\mathrm{T}}\Delta {\mathbf{u}}^0-\Delta {\mathbf{L}}^0\right)\end{array}} $$

where Δ**F**^0^ is the prestress applied through the actuator length change Δ**L**^0^ and Δ**u**^0^ is the corresponding change of node positions from the input geometry.

When the external load is applied, equilibrium and compatibility constraints are expressed as

17 $$ \mathbf{K} \mathbf{u}=\mathbf{P}, $$

where $\mathbf{K}\in {\mathrm{\mathbb{R}}}^{n^f\times {n}^f}$ is the structure stiffness matrix, $\mathbf{u}\in {\mathrm{\mathbb{R}}}^{n^f\times 1}$ is the displacement vector, and $\mathbf{P}\in {\mathrm{\mathbb{R}}}^{n^f\times 1}$ is the total external load vector which here includes the effect of actuation. For a pin-jointed structure and excluding geometric stiffness contribution, the stiffness matrix **K** in global coordinates is given by

18 $$ \mathbf{K}=\mathbf{A}\overline{\mathbf{K}}{\mathbf{L}}^{-1}{\mathbf{A}}^{\mathrm{T}}. $$

All terms in (18) have been defined in Section 3 including the diagonal element stiffness matrix $\overline{\mathbf{K}}$. Generally, since tensegrity structures have a geometric nonlinear behavior, the stiffness matrix comprises material and geometric stiffness (Zhang and Makoto 2015; Connelly 2002). However, since through actuation it is possible to ensure the cable elements do not slack under the effect of external loading, it is reasonable to assume that material (linear) stiffness is dominant and therefore geometric stiffness is not included in (17).

Since actuation causes a force and a displacement change, it can be effectively incorporated in the external load. The load vector **P** in (17) contains not only the external load **P**^*ext*^ but also the equivalent load caused by actuation

19 $$ \mathbf{P}={\mathbf{P}}^{ext}+{\mathbf{P}}^{act}, $$

where **P**^*act*^ is denoted as “actuator load.” **P**^*act*^ is computed as

20 $$ {\mathbf{P}}^{act}=\mathbf{A}\overline{\mathbf{K}}{\mathbf{L}}^{-1}\Delta \mathbf{L}. $$

Since the actuators are installed in series with the elements, Δ**L** can be thought of as a lack of fit. The change of forces and displacements caused by the actuator is equivalent to that caused by an external load parallel to the axis of the corresponding element and applied to its end nodes, which is expressed by the term $\mathbf{A}\overline{\mathbf{K}}{\mathbf{L}}^{-1}\Delta \mathbf{L}$.

Equilibrium and compatibility constraints for the noncontrolled state under permanent load are

21 $$ {\mathbf{Ku}}^P={\mathbf{P}}^{permanent}, $$

22 $$ {\mathbf{F}}^P=\overline{\mathbf{K}}{\mathbf{L}}^{-1}{\mathbf{A}}^{\mathrm{T}}{\mathbf{u}}^P, $$

where **P**^*permanent*^ denotes the permanent load including dead load (**P**^*dead*^) and structure self-weight (**P**^*self*^). In the controlled state, the actuator length change Δ**L**^*P*^ is applied to counteract the effect of **P**^*permanent*^, and thus equilibrium and compatibility constraints are

23 $$ {\mathbf{Ku}}^{P\_C}={\mathbf{P}}^P, $$

24 $$ {\mathbf{F}}^{P\_C}=\overline{\mathbf{K}}{\mathbf{L}}^{-1}\left({\mathbf{A}}^{\mathrm{T}}{\mathbf{u}}^{P\_C}-\Delta {\mathbf{L}}^P\right), $$

where

25 $$ {\mathbf{P}}^P={\mathbf{P}}^{permanent}+\mathbf{A}\overline{\mathbf{K}}{\mathbf{L}}^{-1}\Delta {\mathbf{L}}^P. $$

Note that **u**^*P_C*^ and **F**^*P_C*^ are controlled displacements and forces (hence the superscript contains *C*) that result from the combined effect of **P**^*permanent*^ and Δ**L**^*P*^. In other words, **u**^*P_C*^ = **u**^*P*^ + Δ**u**^*P*^ and **F**^*P_C*^ = **F**^*P*^ + Δ**F**^*P*^ where **u**^*P*^ are the node displacements and **F**^*P*^ the element forces caused by **P**^*permanent*^ while Δ**u**^*P*^ is the displacement correction and Δ**F**^*P*^ the force correction caused by Δ**L**^*P*^ (see Fig. 4). For clarity, the controlled element force is obtained as the product of the element stiffness $\overline{\mathbf{K}}{\mathbf{L}}^{-1}$ by the element elastic deformation **e** = **e**^*t*^ − *Δ***L** in which **e**^*t*^ = **A**^T^**u** is the total element deformation. The terms **u**^*P*^, **u**^*P_C*^, **F**^*P*^, **F**^*P_C*^, and Δ**L**^*P*^ are optimization variables. Once a solution is obtained, displacement and force corrections required under the permanent load are computed as ∆**u**^*P*^ = **u**^*P_C*^-**u**^*P*^ and Δ**F**^*P*^ = **F**^*P_C*^- **F**^*P*^, respectively.

Similarly, equilibrium and compatibility constraints for the noncontrolled state under live load **P**^*live*^ are

26 $$ {\mathbf{Ku}}^L={\mathbf{P}}^{live}, $$

27 $$ {\mathbf{F}}^L=\overline{\mathbf{K}}{\mathbf{L}}^{-1}{\mathbf{A}}^{\mathrm{T}}{\mathbf{u}}^L. $$

In the controlled state, the actuator length change Δ**L**^*L*^ is applied to counteract the effect of live load, and thus equilibrium and compatibility constraints are

28 $$ {\mathbf{Ku}}^{L\_C}={\mathbf{P}}^L, $$

29 $$ {\mathbf{F}}^{L\_C}=\overline{\mathbf{K}}{\mathbf{L}}^{-1}\left({\mathbf{A}}^{\mathrm{T}}{\mathbf{u}}^{L\_C}-\Delta {\mathbf{L}}^L\right). $$

where

30 $$ {\mathbf{P}}^L={\mathbf{P}}^{live}+\mathbf{A}\overline{\mathbf{K}}{\mathbf{L}}^{-1}\Delta {\mathbf{L}}^L. $$

Controlled displacements **u**^*L_C*^ and forces **F**^*L* _ *C*^ result from the combined effect of **P**^*live*^ and Δ**L**^*L*^. The terms **u**^*L*^, **u**^*L_C*^, **F**^*L*^, **F**^*L_C*^, and Δ**L**^*L*^ are optimization variables. Once a solution is obtained, displacement and force corrections required under the live load are computed as Δ**u**^*L*^ = **u**^*L_C*^-**u**^*L*^ and Δ**F**^*L*^ = **F**^*L_C*^- **F**^*L*^, respectively.

### Ultimate limit state (ULS) constraints

#### Element stress and buckling constraints

Ultimate limit state (ULS) constraints ensure that all element forces are controlled within required limits under the worst loading condition in the controlled states (a), (c), and (e) (Section 2.3)

31 $$ \Big\{{\displaystyle \begin{array}{c}{\underset{\_}{\sigma}}^c{\alpha}_i\le {F}_i^{\psi}\le {\overline{\sigma}}^c{\alpha}_i,\kern0.75em \mathrm{if}\ i\in {S}_{cable},\forall \psi \in \left\{a,c,e\right\}\\ {}{\underset{\_}{\sigma}}^s{\alpha}_i\le {F}_i^{\psi}\le {\overline{\sigma}}^s{\alpha}_i,\kern0.75em \mathrm{if}\ i\in {S}_{strut},\forall \psi \in \left\{a,c,e\right\}\\ {}-{F}_i^b\le {F}_i^{\psi },\kern3.5em \mathrm{if}\ i\in {S}_{strut},\forall \psi \in \left\{a,c,e\right\}\end{array}}, $$

where *S*_*cable*_ and *S*_*strut*_ denote the index set for cable and strut elements, respectively. For clarity, in state (a), **F**^*ψ*^ = **ΔF**^0^(*ψ* = *a*); in state (c), **F**^*ψ*^ = **ΔF**^0^ + **F**^*P* _ *C*^(*ψ* = *c*); and in state (e), **F**^*ψ*^ = **ΔF**^0^ + **F**^*P* _ *C*^ + **F**^*L* _ *C*^(*ψ* = *e*). Typically, state (e) of phase I adaptation is the most demanding control condition since the total element force is the sum of prestress Δ**F**^0^, controlled forces under permanent load **F**^*P* _ *C*^, and live load **F**^*L* _ *C*^. ${\underline{\sigma}}^c$ and ${\overline{\sigma}}^c$ are lower and upper bound, respectively, for the admissible stress in cable elements; ${\underline{\sigma}}^s$ and ${\overline{\sigma}}^s$ are lower and upper bound, respectively, for the admissible stress in strut elements. For stable equilibrium, cables must be in tension under any load condition. The lower bound ${\underline{\sigma}}^c$cannot be smaller than zero, and thus it is set to ${\underline{\sigma}}^c=\zeta {\overline{\sigma}}^c$ where *ζ* is a small positive value. ${\mathbf{F}}^b\in {\mathrm{\mathbb{R}}}^{n^e\times 1}$ is the Euler buckling load vector for strut elements. To reduce optimization complexity, strut elements are assumed to have a circular hollow section with a wall thickness *t* proportional to the external radius *R*^*e*^, i.e., *t* = *γR*^*e*^(*γ* is constant). It follows that the internal radius is *R*^*i*^ = *R*^*e*^(1 − *γ*). This way, the Euler buckling load for a strut element can be expressed as a function of its cross-sectional area as

32 $$ {F}_i^b=\frac{\pi E{\alpha}_i^2\left(1+{\lambda}^2\right)}{4{L}_i^2\left(1-{\lambda}^2\right)},\forall i\in {S}_{strut}, $$

where *λ* = 1 − *γ*. Element cross-sectional areas are constrained between a lower (${\alpha}_{\mathrm{min}}^c$ and ${\alpha}_{\mathrm{min}}^s$) and an upper (${\alpha}_{\mathrm{max}}^c$ and ${\alpha}_{\mathrm{max}}^s$) bound to account for feasible construction and commercial availability

33 $$ \Big\{{\displaystyle \begin{array}{c}{\alpha}_{\mathrm{min}}^c\le {\alpha}_i\le {\alpha}_{\mathrm{max}}^c,\kern0.5em \mathrm{if}\ i\in {S}_{cable}\\ {}{\alpha}_{\mathrm{min}}^s\le {\alpha}_i\le {\alpha}_{\mathrm{max}}^s,\kern0.5em \mathrm{if}\ i\in {S}_{strut}\end{array}}. $$

#### Fail-safe constraints

Fail-safe constraints ensure that the structure can take the worst loading condition without the contribution of the active system in the noncontrolled states (b), (d), and (f) (Section 2.3)

34 $$ \left\{\begin{array}{c}{\underset{\_}{\sigma}}^c{\alpha}_i\le {F}_i^{\psi}\le {\overline{\sigma}}^c{\alpha}_i,\kern0.5em \mathrm{if}\ i\in {S}_{cable},\kern0.5em \forall \psi \in \left\{b,d,f\right\}\\ {}{\underset{\_}{\sigma}}^s{\alpha}_i\le {F}_i^{\psi}\le {\overline{\sigma}}^c{\alpha}_i,\kern1.00em \mathrm{if}\ i\in {S}_{strut},\kern0.5em \forall \psi \in \left\{b,d,f\right\}.\\ {}\begin{array}{c}-{F}_i^b\le {F}_i^{\psi },\kern2.5pc \mathrm{if}\ i\in {S}_{strut},\kern0.5em \forall \psi \in \left\{b,d,f\right\}\end{array}\end{array}\right. $$

For clarity, in state (b), **F**^*ψ*^ = **ΔF**^0^ + **F**^*P*^(*ψ* = *b*); in state (d), **F**^*ψ*^ = Δ**F**^0^ + **F**^*P* _ *C*^ + **F**^*L*^(*ψ* = *d*); and in state (d), **F**^*ψ*^ = **ΔF**^0^ + **F**^*P* _ *C*^ + **ΔF**^*L*^(*ψ* = *f*). Typically, state (d) of phase I adaptation is the most demanding noncontrolled condition since the total element force is the sum of prestress Δ**F**^0^, controlled force under permanent load **F**^*P* _ *C*^_,_ and noncontrolled force under live load **F**^*L*^. Although formally identical, (31) and (34) are conceptually different. Equation (31) ensures that cables do not slack and stress and buckling limits are not exceeded during control (states (a), (c), (e)). Instead, through (34), cables are prevented to slack, and stress and buckling limits are met without the contribution of the active system (states (b), (d), (f)). Therefore, the structure load-carrying capacity is not compromised in case of a power outage and concurrent occurrence of a strong loading event.

### Serviceability limit state (SLS) constraints

Deflection constraints are implemented by setting bounds *u*^limit^ on displacements of selected controlled nodes

35 $$ -{u}^{\mathrm{limit}}\le {u}_i^{\psi}\le {u}^{\mathrm{limit}},\forall i\in {S}_{cdof},\forall \psi \in \left\{c,e\right\}. $$

where ${u}_i^{\psi }$ is the displacement of the *i*^th^ degree of freedom and *S*_*cdof*_ is a set of indices corresponding to the controlled degrees of freedom *cdof* (i.e., controlled nodes). The *cdofs* are input to the design process. Depending on the structure layout, *cdofs* can be selected so that the structure shape is controlled to meet typical deflection limits. For clarity, in state (c), **u**^*ψ*^ = **Δu**^0^ + **u**^*P* _ *C*^(*ψ* = *c*) and in state (e) **u**^*ψ*^ = **Δu**^0^ + **u**^*P* _ *C*^ + **u**^*L* _ *C*^(*ψ* = *e*). Recalling the adaptation phases defined in Section 2.3, under the action of permanent load (phase 0), displacements are reduced practically to zero which is equivalent to the effect of pre-cambering (state (c)). In this case, *u*^limit^ is denoted with *u*^*SLS0*^ which is set to zero or a very small value depending on requirements. During service, under the action of the live load (state (e)), *u*^limit^ is denoted with *u*^*SLS*^ which is set to typical deflection limits.

### Actuator control command constraints

 Constraints for actuator commands are set to avoid large length changes which might be impractical. Also, since an actuator is assumed to be installed in series with the element, the length change is limited to a fraction of the hosting element length. The actuator length change Δ**L** is constrained by

36 $$ -\varDelta {L}_{\mathrm{limit}}{n}_i\le \varDelta {L}_i^{\psi}\le \varDelta {L}_{\mathrm{limit}}{n}_i,\forall i,\forall \psi \in \left\{a,c,e\right\}, $$

where Δ*L*_limit_ is the prescribed limit and *n*_*i*_ is the actuator position binary variable defined in Section 4.1. When *n*_*i*_ = 1, the *i*^*th*^ element is equipped with an actuator and otherwise *n*_*i*_ = 0. For clarity, in state (a) **ΔL**^*ψ*^ = **ΔL**^0^; in state (c), **ΔL**^*ψ*^ = **ΔL**^0^ + **ΔL**^*P*^(*ψ* = *c*); and in state (e), **ΔL**^*ψ*^ = **ΔL**^0^ + **ΔL**^*P*^ + **ΔL**^*L*^(*ψ* = *e*). Concerning the prestress state, to ensure that the displacement Δ**u**^0^ caused by the initial actuator length Δ**L**^0^ is small, the limit for Δ**L**^0^, denoted as $\Delta {L}_{\lim \mathrm{it}}^0$, is set to 10% of Δ*L*_limit_. Large variation from the input geometry caused by prestressing is usually undesired.

Under the permanent load and for the ultimate limit state, actuator control commands must be identical for load cases with an identical load factor

37 $$ \Delta {L}_i^P=\Delta {L}_j^P,\mathrm{if}\kern0.5em {\delta}_i={\delta}_j, $$

where *δ*_*i*_ denotes the load factor for the permanent load of the *i*^*t*h^ load case.

It is generally preferable to operate an adaptive structure with a low number of actuators to minimize control system complexity. Equation (38) is employed to limit the total number of actuators to *n*^*a*^ which can be assigned depending on requirements

38 $$ \sum \limits_i^{n^e}{n}_i\le {n}^a. $$

### Auxiliary constraints

#### Actuator embodied energy auxiliary constraints

The actuation system embodied energy (14) is assumed to be a linear function of the actuator force capacity $\tilde{\mathbf{F}}$ (see Section 4.1). The actuators are assumed to be installed in series with the elements. Therefore, the actuator force capacity must be equal to or larger than the maximum force that the element is subjected to, across all load cases. Since the actuator position are not known, an auxiliary constraint must be set to relate the actuator force capacity with the force of the element onto which the actuator will be installed

39 $$ -{\tilde{F}}_i\le {F}_i^{\psi}\le {\tilde{F}}_i,\forall i,\forall \psi \in \left\{a,b,c,d,e,f\right\}, $$

40 $$ {\tilde{F}}_{\mathrm{min}}\le {\tilde{F}}_i\le {\tilde{F}}_{\mathrm{max}},\forall i, $$

where ${F}_i^{\psi }$ denotes the *i*^th^ element force in a particular state as done in Section 4.3. Equation (39) must be satisfied for all load cases to ensure that all element forces are within [−$\tilde{F}$, $\tilde{F}$_*i*_]. $\tilde{F}$_min_ and $\tilde{F}$_max_ are lower and upper bounds, respectively, on the actuator force capacity which can be set to match commercial availability.

#### Load actuation threshold (*LAT*) auxiliary constraints

Embodied and operational energy minimization problems are coordinated through an auxiliary variable called load activation threshold (*LAT*). *LAT* is the lowest intensity live load, denoted as ${\mathbf{P}}_{LAT}^{live}$, that causes a violation of stress and/or displacement limits during service (Senatore et al. 2019). That is when the live load is smaller than ${\mathbf{P}}_{LAT}^{live}$, stress and displacement limits are satisfied entirely through passive load-bearing capacity; instead, when the live load is larger than ${\mathbf{P}}_{LAT}^{live}$, active control is needed to meet required limit states. It is convenient to express *LAT* in percentage terms as ${\mathbf{P}}_{LAT}^{live}= LAT\cdot {\mathbf{P}}_d^{live}$ where ${\mathbf{P}}_d^{live}$ is the maximum expected live load for the SLS case (i.e., excluding load factors). Note that stress and buckling limits for the ultimate limit state (ULS) are satisfied under all load cases without the contribution of the active system through (34) (fail-safe constraints). Therefore, *LAT* applies only to the serviceability limit state (SLS) for deflection limits.

Two auxiliary constraints are added to enforce the condition that displacement limits are met without the contribution of the active system for any live load event of intensity smaller than ${\mathbf{P}}_{LAT}^{live}$

41 $$ {\mathbf{Ku}}_{LAT}^L={\mathbf{P}}_{LAT}^{live}, $$

42 $$ -{u}^{SLS}\le {u}_{i, LAT}^L\le {u}^{SLS},\forall i\in {S}_{cdof}, $$

where ${\mathbf{u}}_{LAT}^L$ is the displacement caused by ${\mathbf{P}}_{LAT}^{live}$ which is added to the optimization variables. Equation (42) keeps *cdof* displacements under ${\mathbf{P}}_{LAT}^{live}$ within serviceability limits, which thus ensures that SLS is satisfied without contribution of the active system for any live load event of intensity smaller than ${\mathbf{P}}_{LAT}^{live}$.

### Embodied energy minimization, full model formulation (MINLP)

Embodied energy minimization is formulated as a mixed-integer nonlinear programming problem (MINLP). Objective function together with all state and auxiliary constraints (Sections 4.1 to 4.6) are given in Table 1.

**Table 1:** Embodied energy minimization formulation

$$ {\displaystyle \begin{array}{c}\min \\ {}\mathrm{x}\end{array}} $$	${E}_{embd}=\sum \limits_{i=1}^{n^e}{\alpha}_i{L}_i{\rho}_i{e}_i^e+c\sum \limits_{i=1}^{n^e}{F}_i{n}_i{e}_i^a$		Objective function
s.t.			
	$$ {\displaystyle \begin{array}{c}\mathbf{A}\Delta {\mathbf{F}}^0=\mathbf{0}\\ {}\Delta {\mathbf{F}}^0=\overline{\mathbf{K}}{\mathbf{L}}^{-1}\left({\mathbf{A}}^{\mathrm{T}}\Delta {\mathbf{u}}^0-\Delta {\mathbf{L}}^0\right)\end{array}} $$		Prestress
	${\mathbf{Ku}}_{jL}^{P\_C}={\mathbf{P}}_{jl}^P$	∀*j*, ∀ *l*	Equilibrium constraints
	${\mathbf{Ku}}_{jL}^{L\_C}={\mathbf{P}}_{jl}^L$	∀*j*, ∀ *l*	
	${\mathbf{Ku}}_{jL}^P={\mathbf{P}}_{jl}^{permanent}$	∀*j*, ∀ *l*	
	${\mathbf{Ku}}_{jL}^L={\mathbf{P}}_{jl}^{live}$	∀*j*, ∀ *l*	
	$$ {\mathbf{Ku}}_{LAT}^L={\mathbf{P}}_{LAT}^{live} $$		
	${\mathbf{F}}_{jl}^{P\_C}=\overline{\mathbf{K}}{\mathbf{L}}^{-1}\left({\mathbf{A}}^{\mathrm{T}}{\mathbf{u}}_{jl}^{P\_C}\mathbf{\Delta }{\mathbf{L}}_{jl}^P\right)$	∀*j*, ∀ *l*	
	${\mathbf{F}}_{jl}^{L\_C}=\overline{\mathbf{K}}{\mathbf{L}}^{-1}\left({\mathbf{A}}^{\mathrm{T}}{\mathbf{u}}_{jl}^{L\_C}\mathbf{\Delta }{\mathbf{L}}_{jl}^L\right)$	∀*j*, ∀ *l*	
	${\mathbf{F}}_{jl}^P=\overline{\mathbf{K}}{\mathbf{L}}^{-1}{\mathbf{A}}^{\mathrm{T}}{\mathbf{u}}_{jl}^P$	∀*j*, ∀ *l*	
	${\mathbf{F}}_{jl}^L=\overline{\mathbf{K}}{\mathbf{L}}^{-1}{\mathbf{A}}^{\mathrm{T}}{\mathbf{u}}_{jl}^L$	∀*j*, ∀ *l*	
	${\underset{\_}{\sigma}}^c{\alpha}_i\le {F}_{ijl}^{\varPsi }{\le}_{\sigma}^{-c}{\alpha}_i$	∀*i* ∈ *S*_*cable*_, ∀ *j*, ∀ *l*, ∀_*Ψ*∈_{*a*, *c*, *e*}	Element stress and buckling constraints
	${\underset{\_}{\sigma}}^s{\alpha}_i\le {F}_{ijl}^{\varPsi }{\le}_{\sigma}^{-s}{\alpha}_i$	∀*i* ∈ *S*_*strut*_, ∀ *j*, ∀ *l*, ∀_*Ψ*∈_{*a*, *c*, *e*}	
	$-{F}_i^b\le {F}_{ijl}^{\varPsi }$	∀*i* ∈ *S*_*strut*_, ∀ *j*, ∀ *l*, ∀_*Ψ*∈_{*a*, *c*, *e*}	
	${\underset{\_}{\sigma}}^c{\alpha}_i\le {F}_{ijl}^{\varPsi }{\le}_{\sigma}^{-c}{\alpha}_i$	∀*i* ∈ *S*_*cable*_, ∀ *j*, ∀ *l*, ∀_*Ψ*∈_{*b*, *d*, *f*}	Fail-safe constraints
	${\underset{\_}{\sigma}}^s{\alpha}_i\le {F}_{ijl}^{\varPsi }{\le}_{\sigma}^{-s}{\alpha}_i$	∀*i* ∈ *S*_*strut*_, ∀ *j*, ∀ *l*, ∀_*Ψ*∈_{*b*, *d*, *f*}	
	$-{F}_i^b\le {F}_{ijl}^{\varPsi }$	∀*i* ∈ *S*_*strut*_, ∀ *j*, ∀ *l*, ∀_*Ψ*∈_{*b*, *d*, *f*}	
	$-{F}_i\le {F}_{ijl}^{\varPsi}\le {F}_i$	∀*i*, ∀ *j*, ∀ *l*, ∀_*Ψ*_	Auxiliary constraints for actuator embodied energy
	*F*_min_ ≤ *F*_i_ ≤ *F*_max_	∀*i*	
	$-{u}^{SLSO}\le {u}_{ijl}^{\varPsi}\le {u}^{SLSO}$	∀*i* ∈ *S*_*cdof*_, ∀ *j*, *l* = {*SLS*}, *Ψ* = *c*	Displacement constraints
	$-{u}^{SLS}\le {u}_{ijl}^{\varPsi}\le {u}^{SLS}$	∀*i* ∈ *S*_*cdof*_, ∀ *j*, *l* = {*SLS*}, *Ψ* = *e*	
	$-{u}^{SLS}\le {u}_{ijl,L\ AT}^L\le {u}^{SLS}$	∀*i* ∈ *S*_*cdof*_, ∀ *j*, *l* = {*SLS*}	
	$-\Delta {L}_{\mathrm{limit}}^0{n}_i\le \Delta {L}_i^{\varPsi}\le \Delta {L}_{\mathrm{limit}}^0{n}_i$	∀*i*, *Ψ* = *a*	Actuator layout constraints
	$-\Delta {L}_{\mathrm{limit}}^0{n}_i\le \Delta {L}_{ijl}^{\varPsi}\le \Delta {L}_{\mathrm{limit}}{n}_i$	∀*i*, ∀ *j*, ∀ *l*, ∀_*Ψ*∈_(*c*, *e*)	
	$\Delta {L}_{il}^P=\Delta {L}_{jl}^P,\mathrm{if}\ {\delta}_i={\delta}_j$	*l* = {*ULS*	
	$$ \sum \limits_i^{n^e}{n}_i\le {n}^a $$		
	*n*_*i*_ ∈ {0, 1}	∀*i*	
	${\alpha}_{\mathrm{min}}^c\le {\alpha}_i\le {\alpha}_{\mathrm{max}}^c$	∀*i* ∈ *S*_*cable*_	Bounds for element cross-section areas
	${\alpha}_{\mathrm{min}}^s\le {\alpha}_i\le {\alpha}_{\mathrm{max}}^s$	∀*i* ∈ *S*_*strut*_	

The vector $\mathbf{X}=\left(\boldsymbol{\upalpha}, \mathbf{n},\tilde{F},\varDelta {\mathbf{F}}^0,\varDelta {\mathbf{u}}^0,\varDelta {\mathbf{L}}^0,{\mathbf{F}}^P,{\mathbf{F}}^{P\_C},{\mathbf{u}}^P,{\mathbf{u}}^{P\_C},\varDelta {\mathbf{L}}^P,{\mathbf{F}}^L,{\mathbf{F}}^{L\_C},{\mathbf{u}}^L,{\mathbf{u}}^{L\_C},{\mathbf{u}}_{LAT}^L,\varDelta {\mathbf{L}}^L\right)$ collates all optimization variables including design and state variables which are also listed in Table 2. The design variables are element cross-sectional areas **α** and the vector of binary variables **n** for the actuator positions. The actuator force capacity $\tilde{F}$ is an auxiliary variable that is employed to compute the actuation system embodied energy as explained in Sections 4.1 and 4.6. The state variables are:

- Prestress state (*Δ***F**^0^, *Δ***u**^0^, *Δ***L**^0^) under no external load
- Noncontrolled state (**F**^*P*^, **u**^*P*^) under **P**^*permanent*^
- Controlled state (**F**^*P* _ *C*^, **u**^*P* _ *C*^, *Δ***L**^*P*^) under **P**^*permanent*^
- Noncontrolled state (**F**^*L*^, **u**^*L*^) under **P**^*live*^
- Controlled state (**F**^*L* _ *C*^, **u**^*L* _ *C*^, *Δ***L**^*L*^) under **P**^*live*^
- Displacement ${\mathbf{u}}_{LAT}^L$ under load activation threshold ${\mathbf{P}}_{LAT}^{live}$

**Table 2:** Embodied energy optimization variables

Continuous variable					
*V*	**α**	$\tilde{\mathbf{F}}$			
*N*	*n*^*e*^	*n*^*e*^			
*V*	Δ**F**^0^	Δ**u**^0^	Δ**L**^0^		
*N*	*n*^*e*^	*n*^*f*^	*n*^*e*^		
*V*	**F**^*P*^	**u**^*P*^	**F**^*P* _ *C*^	**u**^*P* _ *C*^	Δ**L**^*P*^
*N*	*n*^*e*^(*n*^*p*^ + 1)	*n*^*f*^(*n*^*p*^ + 1)	*n*^*e*^(*n*^*p*^ + 1)	*n*^*f*^(*n*^*p*^ + 1)	*n*^*e*^(*n*^*p*^ + 1)
*V*	**F**^*L*^	**u**^*L*^	**F**^*L* _ *C*^	**u**^*L* _ *C*^	*Δ***L**^*L*^
*N*	2*n*^*e*^*n*^*p*^	2*n*^*f*^*n*^*p*^	2*n*^*e*^*n*^*p*^	2*n*^*f*^*n*^*p*^	2*n*^*e*^*n*^*p*^
*V*	${\mathbf{u}}_{LAT}^L$				
*N*	2*n*^*f*^*n*^*p*^				
Binary variable					
*V*	**n**				
*N*	*n*^*e*^				

Unless otherwise indicated, the indices *i*, *j*, *l*, *ψ* iterate over *n*^*e*^ structural elements, *n*^*p*^ load cases,{*ULS*, *SLS*} load combination cases, and {*a*, *b*, *c*, *d*, *e*, *f*} states, respectively. Both *ULS* and *SLS* are considered to ensure that all constraints are satisfied for the corresponding limit state. Note that prestress-related terms in ${F}_{ijl}^{\psi }$, ${u}_{ijl}^{\psi }$, and $\Delta {L}_{ijl}^{\psi }$ do not iterate on *j* and *l* because the prestress state is unique.

For a structure with *n*^*e*^ elements and *n*^*f*^ free degrees of freedom that is subjected to *n*^*p*^ load cases, the total number of optimization variables is

43 $$ {\displaystyle \begin{array}{l}{n}_c^v={n}^e\left(9{n}^p+7\right)+{n}^f\left(8{n}^p+3\right)\\ {}{n}_b^v={n}^e\end{array}} $$

where ${n}_c^v$ and ${n}_b^v$ are the number of continuous and binary variables, respectively. Force and displacement variables related to the live load are doubled because USL and SLS are accounted for. Instead, only an extra vector is added to force and displacement variables under permanent load for SLS because all load factors are always set identical. The embodied energy minimization process is summarized in Table 3.

**Table 3:**
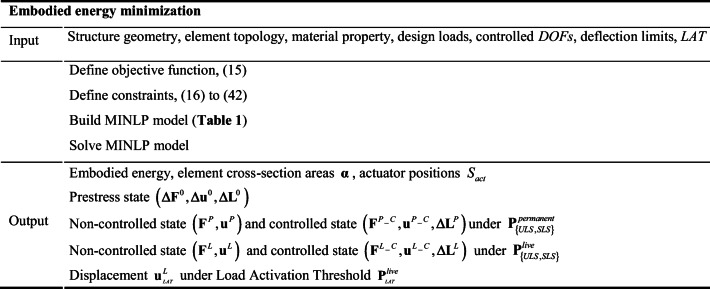
Embodied energy minimization summary

Given a load activation threshold (*LAT*), this process produces an adaptive tensegrity structure which is an optimal configuration in embodied energy cost terms. The set of indices *S*_*act*_ for the actuator positions are extracted from **n** once the solution is obtained. The structure is designed to resist adequately all loading events of intensity lower than *LAT* without the contribution of the active system. For loading events larger than *LAT* up to the design load, although stress and buckling limits are satisfied without contribution of the active system (fail-safe), deflections must be reduced to satisfy serviceability.

## Operational energy minimization

Element sizing and actuator layout (**α**, *S*_*act*_), as well as prestress and controlled state under permanent load obtained through embodied energy minimization, are inputs to the second phase of the design process which is operational energy minimization. The actuator commands to control the structural response under live load for USL and SLS have already been computed through embodied energy minimization. In that case, the actuator commands are part of the state variables which have to be computed to size adequately both structure and actuator layout. The actuator commands that are obtained through embodied energy minimization are one of the many feasible solutions to control the structural response under SLS and ULS load cases but they might not be optimal in operational energy terms. The objective of operational energy minimization is to compute actuator commands to control the structure response during service (SLS) under all loading events larger than *LAT* using minimum energy.

### Control through force and shape influence matrices

Refer to the adaptation phases defined in Section 2.3. When the live load is applied, the structure is in state (d) which is the superposition of prestress state, controlled state under permanent load, and noncontrolled state under live load

44 $$ \left\{\begin{array}{ll}{\mathbf{F}}^{\psi }& ={\boldsymbol{\Delta} \mathbf{F}}^0+{\mathbf{F}}^{P\_C}+{\mathbf{F}}^L\\ {}{\mathbf{u}}^{\psi }& =\Delta {\mathbf{u}}^0+{\mathbf{u}}^{P\_C}+{\mathbf{u}}^L\end{array},\kern0.5em \right.\psi =d. $$

Prestress state (*Δ***F**^0^, *Δ***u**^0^) and controlled state under permanent load (**F**^*P* _ *C*^, **u**^*P* _ *C*^) have been obtained through embodied energy minimization and thus are constants at this stage. The noncontrolled state (**F**^*L*^, **u**^*L*^) under all live load events that are larger than *LAT* is obtained through analysis. In state (d) elements forces satisfy required stress and buckling limits thanks to the fail-safe constraints (Section 4.3.2). However, under loading events larger than *LAT*, displacements must be reduced through shape control to satisfy serviceability. Therefore, in phase I adaptation (from state (c) to (e)), the actuators perform a change of length (*Δ***L**) so that all controlled displacements satisfy deflection limits. For statically indeterminate configurations, a change of shape modifies the element forces; therefore, *Δ***L** must be obtained ensuring that stress and buckling limits are satisfied. After a live load event has occurred, the structure is in state (f)

45 $$ \left\{\begin{array}{l}{\mathbf{F}}^{\psi }={\boldsymbol{\Delta} \mathbf{F}}^0+{\mathbf{F}}^{P\_C}+{\boldsymbol{\Delta} \mathbf{F}}^L\\ {}{\mathbf{u}}^{\psi }=\Delta {\mathbf{u}}^0+{\mathbf{u}}^{P\_C}+{\boldsymbol{\Delta} \mathbf{u}}^L\end{array}\right.,\psi =f. $$

In phase II adaptation (from state (e) to (g)), the actuators perform an identical but opposite change of length (−*Δ***L**) to control the structure back into the default state (g).

Assuming small deformations, a simple way to compute the change of element forces Δ**F** and node displacements *Δ***u** caused by actuator commands Δ**L** is to employ the force and shape influence matrices as defined in (Senatore et al. 2019).

46 $$ {\displaystyle \begin{array}{c}\Delta \mathbf{F}={\mathbf{S}}_f\Delta \mathbf{L}\\ {}\Delta \mathbf{u}={\mathbf{S}}_u\Delta \mathbf{L}\end{array}} $$

Element cross-sectional areas **α** and actuator positions *S*_*act*_ are known at this stage. Therefore, force ${\mathbf{S}}_f\in {\mathrm{\mathbb{R}}}^{n^e\times {n}^a}$ and shape ${\mathbf{S}}_u\in {\mathrm{\mathbb{R}}}^{n^f\times {n}^a}$ influence matrices can be simply computed by collating column-wise the effect on element forces and node displacements, respectively, of a unitary length change of each actuator in turn. Note that for statically determinate configurations, **S**_*f*_ is always a zero matrix because the actuator length changes do not affect element forces (Senatore et al. 2019). Alternative closed-form solutions to compute force and shape influence matrices are given in (Reksowardojo and Senatore 2020). Note that since both **S**_*f*_ and **S**_*u*_ contains *n*^*a*^ columns, the actuator length change dimensions must be reduced accordingly, $\Delta \mathbf{L}\in {\mathrm{\mathbb{R}}}^{n^a\times 1}$. Using the force and shape influence matrices allows expressing the controlled element forces **F**^*L* _ *C*^ and node displacements **u**^*L* _ *C*^ under the live load as a function of Δ**L**

47 $$ {\displaystyle \begin{array}{c}{\mathbf{F}}^{L\_C}={\mathbf{F}}^L+\Delta {\mathbf{F}}^L\\ {}{\mathbf{u}}^{L\_C}={\mathbf{u}}^L+\Delta {\mathbf{u}}^L\end{array}}, $$

where Δ**F**^*L*^ and Δ**u**^*L*^ are given by (46). This way the operational energy minimization problem simplifies because the only primary optimization variable is *Δ***L**^*L*^.

### Objective function

Following (Senatore et al. 2019), the objective function is the minimization of the actuator work that is required for structural adaptation during service. Recalling the live load probability distribution definition given in Section 2.2 and the adaptation phases defined in Section 2.3, the work done by the *i*^th^ actuator for the *k*^th^ occurrence of the *j*^th^ load case ${\mathbf{P}}_{jk}^{live}$ is computed as

48 $$ \Big\{{\displaystyle \begin{array}{c}{W}_{ijk}^I={F}_{ijk}^{\psi}\varDelta {L}_{ijk}^L+\frac{1}{2}\varDelta {F}_{ijk}^L\varDelta {L}_{ijk}^L,\forall i\in {S}_{act},\psi =d\\ {}{W}_{ijk}^{II}={F}_{ijk}^{\psi}\left(-\varDelta {L}_{ijk}^L\right)+\frac{1}{2}\left(-\varDelta {F}_{ijk}^L\right)\left(-\varDelta {L}_{ijk}^L\right),\forall i\in {S}_{act},\psi =f\end{array}} $$

where ${W}_{ijk}^{(I)}$ and ${W}_{ijk}^{(II)}$ are the work share done in phase I and phase II adaptation, ${F}_{ijk}^{\psi }$ is the element force before control in state (d) and (f) (see (44) and (45)), and $\Delta {F}_{ijk}^L$ is the force correction caused by the actuator length change $\Delta {L}_{ijk}^L$. Note that for statically determinate configurations, a shape change through actuation does not cause a change of element forces and thus Δ*F*_*ijk*_ is zero (Senatore et al. 2019).

The sign of the objective function depends on the product between the applied force and the actuator length change. An actuator does work when it extends (positive $\Delta {L}_{ijk}^L$) under compression (negative ${F}_{ijk}^{\psi }$ or $\Delta {F}_{ijk}^L$) and when it contracts (negative $\Delta {L}_{ijk}^L$) under tension (positive ${F}_{ijk}^{\psi }$ or $\Delta {F}_{ijk}^L$). Otherwise, no work is required, and there would be a theoretical gain of energy which is neglected to compute a conservative estimate of the operational energy. Only when the product ${W}_{ijk}^F={F}_{ijk}^{\psi}\Delta {L}_{ijk}^L$ and/or ${W}_{ijk}^{\Delta F}=\frac{1}{2}\Delta {F}_{ijk}^L\Delta {L}_{ijk}^L$ is negative, the absolute value is added to the operational energy

49 $$ {W}_{ijk}=\Big\{{\displaystyle \begin{array}{c}\mid {W}_{ijk}^F\mid, \kern4em \mathrm{if}\ {W}_{ijk}^F<0,{W}_{ijk}^{\varDelta F}\ge 0\\ {}\mid {W}_{ijk}^{\varDelta F}\mid, \kern3.75em \mathrm{if}\ {W}_{ijk}^F\ge 0,{W}_{ijk}^{\varDelta F}<0\\ {}\mid {W}_{ijk}^F\mid +\mid {W}_{ijk}^{\varDelta F}\mid, \kern1.00em \mathrm{if}\ {W}_{ijk}^F<0,{W}_{ijk}^{\varDelta F}<0\\ {}0,\kern6em \mathrm{if}\ {W}_{ijk}^F\ge 0,{W}_{ijk}^{\varDelta F}\ge 0\end{array}},\forall i\in {S}_{act},\psi =d. $$

Equation (49) holds for phase I and phase II work shares, which for brevity are both denoted with *W*_*ijk*_. Summing the work for phase I and phase II adaptation, the total operational energy required for each occurrence of the live load that is larger than the load activation threshold is

50 $$ {E}_{op}=\sum \limits_{i\in {S}_{act}}\sum \limits_{j=1}^{n^p}\sum \limits_{k_{LAT}}^{n^d}\frac{\left({W}_{ijk}^I+{W}_{ijk}^{II}\right)\varDelta {t}_{jk}\omega }{\eta }, $$

where *n*^*p*^ is the number of load cases, *n*^*d*^the number of bins of the discretized load probability distribution, and *k*_*LAT*_ is the bin corresponding to *LAT*. The duration of each loading event Δ*t*_*jk*_ is obtained through scaling the expected structure service life with the *k*^th^ occurrence probability for the *j*^th^ load case. The terms *η* and *ω* are mechanical efficiency and working frequency of the actuators, respectively. The mechanical efficiency is specific to the type of actuation technology (Huber et al. 1997). The actuator working frequency is set to the first natural frequency of the structure which is, generally, a conservative assumption to obtain an upper bound of the operational energy (Senatore et al. 2019).

### Optimization constraints

State constraints (e.g., element stress and buckling, displacement, and actuator length change limits) are identical to those formulated for embodied energy minimization (see Sections 4.3, 4.4, and 4.5).

To handle the sign-dependent discontinuity of the objective function ((50)), the terms ${W}_{ijk}^F$ and ${W}_{ijk}^{\Delta F}$ are treated as auxiliary variables, and a set of auxiliary constraints are added to enforce the sign-dependency condition given in (49) for both adaptation phases

51 $$ \Big\{{\displaystyle \begin{array}{c}{W}_{ijk}^{F(I)}\le {F}_{ijk}^{\psi}\varDelta {L}_{ijk}^L,\psi =d\\ {}{W}_{ijk}^{F(II)}\le {F}_{ijk}^{\psi}\left(-\varDelta {L}_{ijk}^L\right),\psi =f\\ {}{W}_{ijk}^{\varDelta F(I)}\le \frac{1}{2}\varDelta {F}_{ijk}^L\varDelta {L}_{ijk}^L\\ {}{W}_{ijk}^{\varDelta F(II)}\le \frac{1}{2}\left(-\varDelta {F}_{ijk}^L\right)\left(-\varDelta {L}_{ijk}^L\right)\\ {}{W}_{ijk}^{F(I)}\le 0,{W}_{ijk}^{F(II)}\le 0\\ {}{W}_{ijk}^{\varDelta F(I)}\le 0,{W}_{ijk}^{\varDelta F(II)}\le 0\end{array}},\forall i\in {S}_{act}. $$

This way the work done by the *i*^th^ actuator for the *k*^th^ occurrence of the *j*^th^ load case ${\mathbf{P}}_{jk}^{live}$ can be expressed as a continuous linear function which is the sum of the auxiliary variables

52 $$ \Big\{{\displaystyle \begin{array}{c}{W}_{ijk}^{(I)}=-\left({W}_{ijk}^{F(I)}+{W}_{ijk}^{\varDelta F(I)}\right),\forall i\in {S}_{act},\psi =d\\ {}{W}_{ijk}^{(II)}=-\left({W}_{ijk}^{F(II)}+{W}_{ijk}^{\varDelta F(II)}\right),\forall i\in {S}_{act},\psi =f\end{array}}. $$

Note that (52) holds for the set of feasible solutions (*Δ***L**^*L*^, **W**^*F*(*I*)^, **W**^Δ*F*(*I*)^, **W**^*F*(*II*)^, **W**^Δ*F*(*II*)^) that satisfy (51) with equality, which includes the minimum energy solution.

### Operational energy minimization, full model formulation (NLP)

Operational energy minimization is formulated as a nonlinear programming problem (NLP). Objective function together with all state and auxiliary constraints (Sections 5.1 to 5.4) are given in Table 4. The vector **X** = (*Δ***L**^*L*^, **W**^*F*(*I*)^, **W**^Δ*F*(*I*)^, **W**^*F*(*II*)^, **W**^Δ*F*(*II*)^) collates the primary optimization variable together with auxiliary variables. All variables are also listed in Table 5.

**Table 4:**
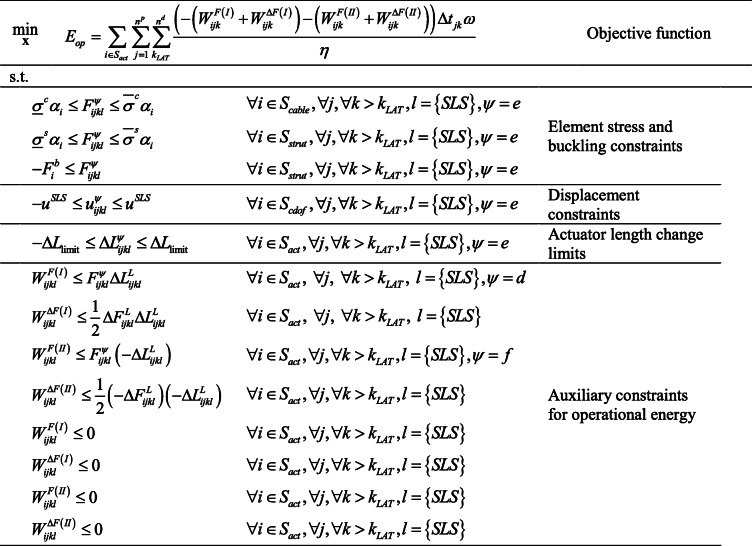
Operational energy minimization formulation

**Table 5:** Operational energy optimization variables

Continuous variable				
*V*	*Δ***L**^*L*^	**W**^*F*(*I*)^	**W**^*F*(*II*)^	**W**^Δ*F*^
*N*	*n*^*a*^*n*^*p*^*n*^*k*^	*n*^*a*^*n*^*p*^*n*^*k*^	*n*^*a*^*n*^*p*^*n*^*k*^	*n*^*a*^*n*^*p*^*n*^*k*^

Unless otherwise indicated, the indices *i*, *j*, and *k* iterates over *n*^*a*^ structural elements, *n*^*p*^ load cases, and all live load occurrences of intensity larger than *LAT* (i.e., ∀*k* > *k*_*LAT*_). Since the operational energy is computed during service, only the SLS load combination case is considered. The index *ψ* indicates the state that applies for a particular constraint. Note that among the terms contained in ${F}_{ijl}^{\psi }$, ${u}_{ijl}^{\psi }$ and $\Delta {L}_{ijl}^{\psi }$, those related to prestress do not iterate on *j* and *l* because the prestress state is unique while those related to permanent load do not iterate on *k* because the probability distribution is defined only for the live load.

The total number of optimization variables is

53 $$ {n}_c^v=4{n}^a{n}^p{n}^k $$

where *n*^*k*^ is the number of live load occurrences of intensity larger than *LAT*. **W**^*F*^variables are doubled to account for phase I and phase II adaptation, while *Δ***L**^*L*^ and **W**^Δ*F*^ are identical in both phases (see Section 2.3). The operational energy minimization process is summarized in Table 6.

**Table 6:**
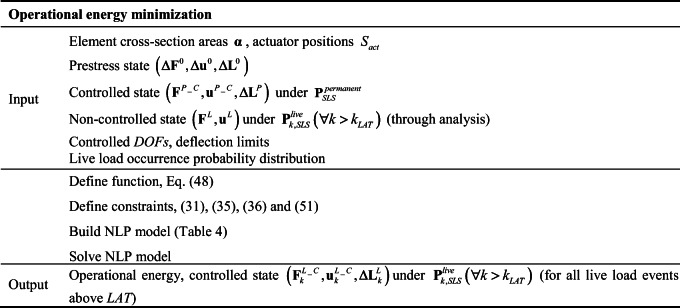
Operational energy minimization summary

## Total energy optimization (TEO)

A flowchart of the total energy minimization process is given in Fig. 2. Embodied and operational energy minimization problems are nested within a univariate optimization process that minimizes the structure total energy—total energy optimization (TEO). The variable of TEO is the load activation threshold (*LAT*). Setting *LAT* to 100% produces stiff and high embodied energy structures that do not require active compensation of displacements during service. On the contrary, setting *LAT* to a low value produces low embodied energy (lightweight) and flexible structures that require active compensation of displacements under low-intensity loading events, which depending on the frequency of occurrence, might require high operational energy during service.

A suitable *LAT* range (discrete values), 0 %  ≤ *LAT* ≤ 100% must be predefined to carry out TEO. For each *LAT*, embodied and operational energy minimization is carried out, and then the minimum total energy configuration is selected as the optimal solution. This methodology can be applied to obtain minimum energy adaptive truss as well as adaptive tensegrity configurations. For tensegrity configurations, cable and strut topology must be defined as part of the input element topology, and the optimal prestress state is included in the output. The total energy minimization process can be summarized as shown in Table 7.

**Table 7:**
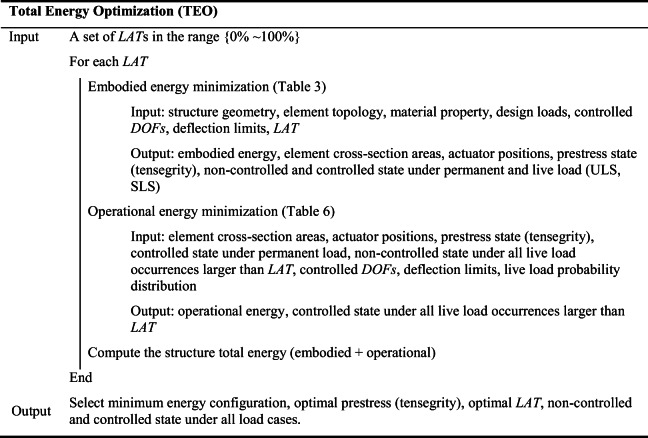
Total energy optimization (TEO) summary

The *LAT* lower bound can be set in reference to the characteristic of the considered load probability distribution. For example, setting *LAT* to a loading event of intensity larger than the mean of the load probability distribution will automatically exclude solutions that require high operational energy, thereby reducing significantly the solution space. It is intuitively clear that the minimum total energy solution is likely to be produced when *LAT* is set to a relatively high value, and thus the active system is employed against loading events with a low probability of occurrence. From experience, typically the optimal *LAT* is located in the range of (50–100%). Using a fine-discretized *LAT* range is likely to produce a better solution but it might require a significantly longer computation time. It is convenient to start with a coarse-discretized *LAT* range and iteratively intensify the search by setting a finer-discretized range that is centered on the optimal *LAT* obtained in the previous iteration. A good first-guess solution is usually obtained by discretizing the *LAT* range (50–100%) in 10 steps. Assuming the first-guess optimal solution is obtained for *LAT* = 70%, subdivide further into 10 steps the *LAT* range (60–80%), and repeat until convergence.

## Numerical examples

**Fig. 5:**
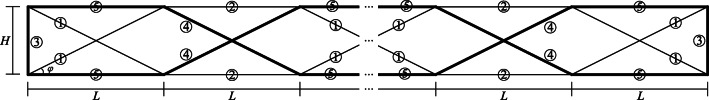
Structure topology pattern, element groups are indicated by labels

TEO is applied to the design of a tensegrity roof and a tensegrity high-rise structure. The two structural configurations under consideration have the same topology pattern that is shown in Fig. 5. Cable and strut elements are represented by thin and thick lines, respectively. This novel tensegrity configuration was obtained through a topology optimization formulation given in (Xu et al. 2018), and its mechanical properties have been studied in (Li et al. 2020).

This system is a class 2 tensegrity structure, according to the definition given in (Skelton and Oliveira 2009). This is a periodic topology pattern that has one self-stress state and no mechanism mode. Considering the topology periodicity and symmetry, elements can be clustered in five groups which are indicated by labels in Fig. 5. Elements of the same group have identical self-stress as given in Table 8.

**Table 8:** Self-stress state

	Cables		Struts		
Element type	①	②	③	④	⑤
Self-stress	1	cos*ϕ*	-sin*ϕ*	-1	-cos*ϕ*

Note: $$ \phi ={\tan}^{-1}\left(\frac{H}{L}\right) $$

### Parameter settings

Strut elements are assumed to have a circular hollow section and to be made of structural steel with a Young modulus *E*_*s*_ = 185 GPa and an admissible stress ${\overline{\sigma}}^s=355$ MPa. Cable elements are assumed to be made of high-strength steel strands with a Young modulus *E*_*c*_ = 206 GPa and an admissible stress ${\overline{\sigma}}^c=1260$ MPa. To maintain a tension state in cable elements under all load cases, the lower bound for the cable stress constraints in (31) and (34) is set to a fraction of the admissible stress ${\underline{\sigma}}^c=\zeta {\overline{\sigma}}^c$ where *ζ* = 0.05. The minimum external radius for strut elements is set to 50 mm, and the wall thickness is set to 10% of the radius. The minimum radius for cable elements is set to 5 mm. For simplicity, all elements and actuators are assumed to be made entirely of steel with a density of 7800 kg/m^3^ and an energy intensity of 36.5 MJ/kg (Hammond and Jones 2008).

Since loading and support conditions are different, the controlled degrees of freedom (*cdofs*) are assigned separately for each case study. The maximum number of actuators that can be assigned is set to *n*^*a*^ = *cdofs* + *s* which is the sum of *cdofs* and the degree of static indeterminacy. This is the minimum number of actuators to control exactly all element forces and *cdof* displacements (Senatore and Reksowardojo 2020). To limit solution space size, the total number of actuators is constrained to *n*^*a*^, i.e., $\sum \limits_i^{n^e}{n}_i={n}^a$ instead of $\sum \limits_i^{n^e}{n}_i\le {n}^a$ as given in the embodied energy minimization formulation (Table 1). Actuators are assumed to be hydraulic with a mechanical efficiency of 0.8 (Huber et al. 1997). Minimum ${\tilde{F}}_{\mathrm{min}}$ and maximum ${\tilde{F}}_{\mathrm{max}}$ actuator force capacity are set to 0 and 1 × 10^5^ kN, respectively (40). The structure service life is set to 50 years. The load probability distribution is discretized with *n*^*d*^ = 50 bins.

### Utilization factors

The terms ${UT}_c^{\mathrm{max}}$ and ${UT}_c^{\mathrm{min}}$ in (54) denote the maximum and minimum utilization ratios, respectively, for cable elements. *F*_*i*_ and ${\overline{\sigma}}^c{\alpha}_i$ are axial and admissible forces for the *i*^th^ element, respectively. If all cable forces are within the admissible stress limit under all load cases, then $0\le {UT}_c^{\mathrm{max}}\le 1$; if all cable elements do not slack under any load case, then $\zeta \le {UT}_c^{\mathrm{min}}\le 1$.

54 $$ \left\{\begin{array}{l}{UT}_c^{\mathrm{max}}=\max \left(\frac{F_i}{{\overline{\sigma}}^c{\alpha}_i}\right)\\ {}{UT}_c^{\mathrm{min}}=\min \left(\frac{F_i}{{\overline{\sigma}}^c{\alpha}_i}\right)\end{array}\right.,i\in {S}_{cable} $$

Similarly, the terms ${UT}_s^{\sigma }$ and ${UT}_s^b$ in (55) denote utilization ratios for strut elements in terms of admissible stress and buckling, respectively

55 $$ \left\{\begin{array}{l}{UT}_s^{\sigma }=\max \left(\left|\frac{F_i}{{\overline{\sigma}}^S{\alpha}_i}\right|\right)\\ {}{UT}_s^b=\max \left(\frac{F_i}{-{F}_i^b}\right)\end{array}\right.,i\in {S}_{strut} $$

where ${F}_i^b$ is the Euler buckling load for the *i*^th^ strut element (*i* ∈ *S*_*strut*_). If all strut forces are within admissible stress and buckling limits under all load cases, then $0\le {UT}_s^{\sigma}\le 1$ and ${UT}_s^b\le 1$.

The term *UT*^*SLS*^ in (56) denotes the ratio between the maximum displacement among the *cdofs* and the deflection limit

56 $$ {UT}^{SLS}=\max \left(\left|\frac{u_i}{u_i^{SLS}}\right|\right),i\in {S}_{cdof} $$

where *u*_*i*_ and ${u}_i^{SLS}$ are displacement and deflection limit for the *i*^*t*h^ *cdof*, respectively. If the displacements of all controlled nodes are within deflection limits under all load cases, then 0 ≤ *UT*^*SLS*^ ≤ 1.

### Benchmark with passive tensegrity and equivalent adaptive truss solutions

The adaptive solution obtained through total energy optimization (TEO) is benchmarked against a passive solution of identical geometry and element topology. The passive solution must be adequately sized so that ULS and SLS are satisfied. In addition, since element forces and node displacements cannot be actively controlled, prestress must be assigned so that the cables do not slack under all load cases. The passive solution is obtained using a similar formulation to that given for embodied energy minimization (Section 4) which is reduced to a continuous nonlinear programming problem (NLP) because the actuator position binary variables are excluded. Optimization variables for the passive design solution are element cross-sectional areas, prestress state, forces, and displacements under permanent and live load. The embodied energy minimization formulation for the passive solution is given in Appendix A. The adaptive tensegrity solution is further benchmarked against an adaptive truss system of identical geometry and topology which has been designed through TEO (Section 6). TEO can be directly applied to truss structures by excluding the prestress state from the variables and the unilaterality condition on elements forces from the constraints (all truss elements can take tension and compression).

TEO has been successfully solved using different algorithms. The best solutions for embodied and operational energy minimization have been obtained using the branch-and-bound algorithm and the interior-point algorithm, respectively, both implemented in Knitro (Nocedal 2006). Further information regarding the variation of solutions obtained through different algorithms is given in Section 7.7.

### Tensegrity roof configuration

#### Dimensions and boundary conditions

**Fig. 6:**
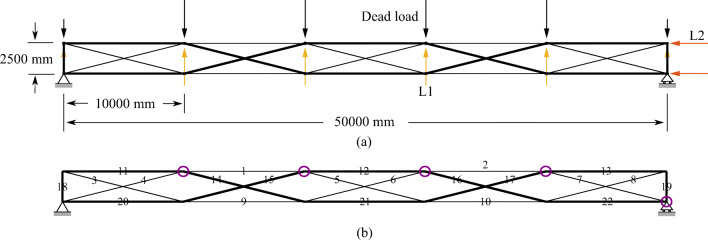
Tensegrity roof: **a** dimensions and loading; **b** element numbering and controlled nodes

Figure 6a shows the dimensions, support, and loading conditions of the tensegrity roof structure considered in this example. The structure has a span of 50 m and a depth of 2.5 m. Figure 6b shows the element numbering and controlled nodes which are indicated by circles. The vertical displacements of the top chord nodes and the horizontal displacement of the roller support are controlled for a total of 5 *cdofs*. The displacement limit for all c*dofs* is set to span/500 = 100 mm. The number of actuators is set to *n*^*a*^ = 5(*cdofs*) + 1(*s*) = 6 (see Section 7.1).

The structure is assumed to be part of a roof system that supports an out-of-plane cover of 10 m. A dead load of magnitude 0.98 kN/m^2^ (100 kg/ m^2^) is applied to the top chord. Two live loads that result from wind action are considered: an uplift load (L1) applied to the top chord and a lateral load (L2). L1 and L2 are distributed loads of magnitude 0.98 kN/m^2^. All loads are appropriately lumped at nodes through the tributary area of the elements. Table 9 gives all design loads and combination cases.

**Table 9:** Summary of load cases, tensegrity roof

Load combination case	Load factor	Permanent load	Load factor	Live load
LC1	1.35	Self-weight + dead load	1.5	–
LC2	0.9	Self-weight + dead load	1.5	L1 = 0.98 kN/m^2^
LC3	1.35	Self-weight + dead load	1.5	L2 = 0.98 kN/m^2^
LC4	0.9	Self-weight + dead load	1.5	L3 = L1 + L2

#### Adaptive vs passive tensegrity

**Fig. 7:**
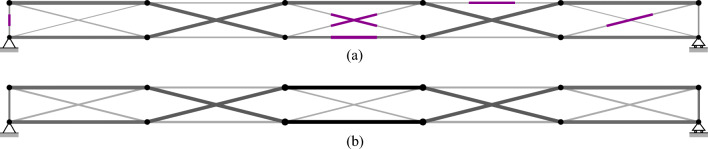
Optimal solutions: **a** adaptive tensegrity roof (ATS) and **b** passive tensegrity roof (PTS)

Figure 7a and Fig. 7b compare the optimal adaptive tensegrity structure (ATS) obtained through TEO (Section 6) and the passive tensegrity structure (PTS) obtained through the embodied energy minimization method given in Appendix A.

**Fig. 8:**
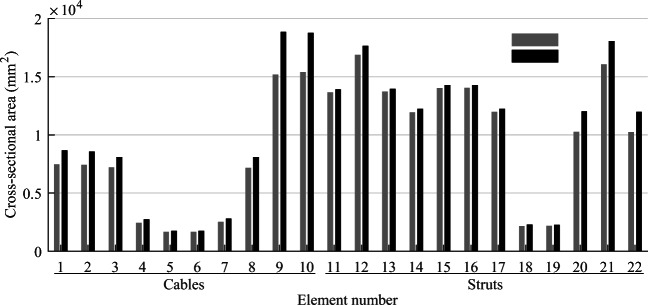
Element cross-sectional areas: adaptive tensegrity roof (ATS) vs passive tensegrity roof (PTS)

The actuators are represented by thick purple line segments placed in the middle of the element. The optimal actuator layout comprises actuators placed on both cables and struts. Element diameters and cross-sectional areas are indicated by line thickness and color shading variation, respectively. The thicker the line, the bigger the diameter, and a darker gray shade indicates a larger cross-sectional area. The element cross-sectional areas are also indicated by the bar chart in Fig. 8. All ATS elements have a smaller cross-sectional area compared to PTS elements. On average the cross-sectional area of ATS elements is 8.9% smaller than that of PTS elements.

**Fig. 9:**
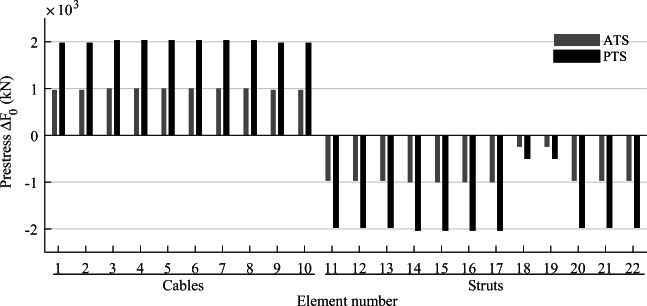
Element prestress: adaptive tensegrity roof (ATS) vs passive tensegrity roof (PTS)

**Fig. 10:**
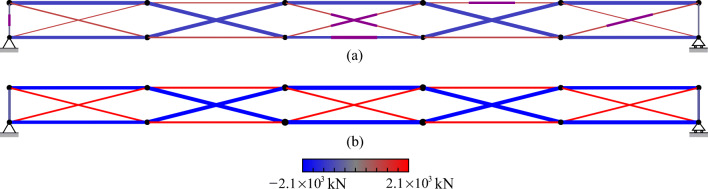
Element prestress: **a** adaptive tensegrity roof (ATS) and **b** passive tensegrity roof (PTS)

Figures 9 and 10 show the element prestress through a bar chart and mapped onto the structure geometry, respectively. As expected, for both ATS and PTS, cables are in tension while the struts are in compression, and in both cases, the prestress state is proportional to the self-stress state (see Table 8). However, since cable elements can be kept in tension through active control, the prestress required in ATS is 50.5% smaller than that required in PTS.

**Fig. 11:**
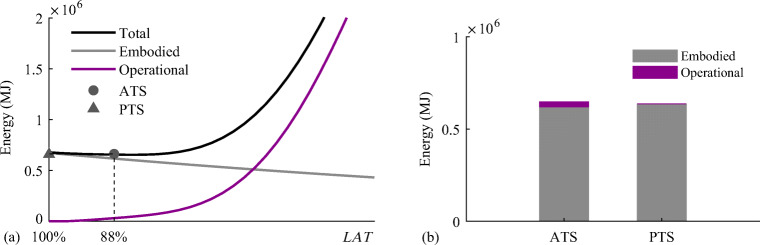
Adaptive tensegrity roof: **a** embodied, operational, and total energy vs *LAT*; **b** energy comparison adaptive (ATS) vs passive (PTS) solution

Figure 11a shows the plot of embodied, operational, and total energy as functions of *LAT*. PTS and ATS are indicated by a triangle and a circle mark, respectively. ATS is obtained for a *LAT* of 88% which is equivalent to a live load of magnitude 0.86 kN/m^2^ (see Table 9). Structural adaptation is required for 1.06 × 10^3^ h under LC2 and LC4 while no adaptation is needed under LC3; thus, the total actuation time is 2.12 × 10^3^ h (approximately 3.0 months over a 50-year service life). The *LAT* for the passive solution (PTS) is 100% because it is sized so that both ultimate and serviceability limit states are satisfied under the worst load case.

Figure 11b compares adaptive and passive configurations in energy cost terms. ATS achieves 1.49% mass savings but no total energy savings (−3.0%) compared to PTS. Mass and embodied energy savings account for the actuation system share. ATS actuation system mass is 6.6% (1.13 × 10^3^ kg) of the total mass (structure + actuators). The maximum force capacity (2.82 × 10^3^ kN) is required for the actuator installed at element 8. The largest length change (−134 mm) is performed by the actuator installed at element 8 under LC1 and LC3. Table 10 gives the optimization metrics for adaptive and passive tensegrity solutions.

**Table 10:** Adaptive tensegrity roof, summary of results

	*LAT*	Mass (kg)	Mass saving	Embodied energy (MJ)	Operational energy (MJ)	Energy saving	Actuation time (hours)	Computation time (seconds)
ATS	88%	1.70 × 10^4^	1.5%	6.21 × 10^5^	2.83 × 10^4^	−3.0%	2.12 × 10^3^	58
PTS	100%	1.74 × 10^4^	–	6.36 × 10^5^	–	–	–	0.21

**Fig. 12:**
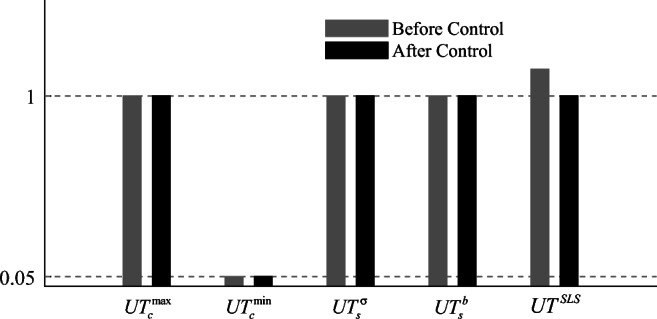
Adaptive tensegrity roof: utilization factors before and after control

Figure 12 shows the utilization factors bar chart (Section 7.2) for ATS before and after control. Cable elements do not slack under any load case, i.e., ${UT}_c^{\mathrm{min}}$ is never smaller than the set lower bound *ζ* = 0.05. For both cables and struts, forces are within admissible limits (${UT}_c^{\mathrm{max}}$, ${UT}_s^{\sigma }$) and strut forces are lower than the buckling limit (${UT}_s^b$). As expected, *UT*^*SLS*^ is larger than 1.0 for loading events of intensity larger.

**Fig. 13:**
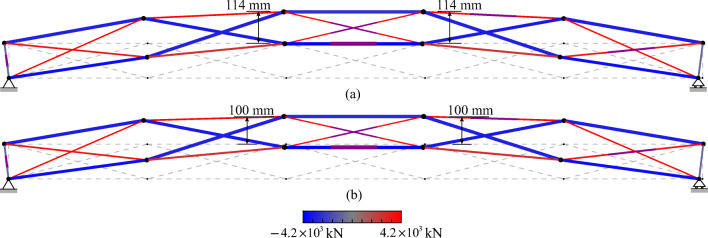
Adaptive tensegrity roof: **a** deformed and **b** controlled shape under LC4

Figure 13 shows ATS deformed shapes before and after control, respectively, under load case LC4. Before control, the two middle node displacements of the top chord exceed deflection limits (100 mm); after control, all node displacements satisfy SLS than *LAT* and reduces to 1.0 through active control.

#### Adaptive tensegrity vs equivalent truss system

**Fig. 14:**
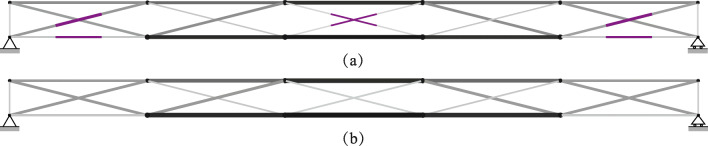
Optimal solutions: **a** adaptive roof truss (AT) and **b** passive roof truss (PT)

TEO is applied to design an equivalent truss structure to benchmark mass and energy savings of the adaptive tensegrity solution. Dimensions, element material, topology, loading, and boundary conditions are identical to the tensegrity roof structure considered in Section 7.4.1. However, all elements of the truss system can carry tension and compression. The optimal passive truss configuration is obtained using the formulation given in Appendix A. The optimal adaptive truss system (AT) and corresponding passive truss system (PT) are shown in Fig. 14. Optimization metrics are given in Table 11.

**Table 11:** Adaptive truss roof, summary of results

	*LAT*	Mass (kg)	Mass saving	Embodied energy (MJ)	Operational energy (MJ)	Energy saving	Actuation time (hours)	Computation time (seconds)
AT	90%	1.20 × 10^4^	7.1%	4.29 × 10^5^	1.11 × 10^4^	4.7%	1.74 × 10^3^	5.53
PT	100%	1.29 × 10^4^	–	4.72 × 10^5^	–	–	–	0.20

Comparing Fig. 14 with Fig. 7 shows that the element cross-sectional distribution of the equivalent truss is significantly different from that of the adaptive tensegrity solution (ATS). AT is obtained for a *LAT* of 90%, which leads to a relatively shorter actuation time compared to ATS. AT actuation system requirements are significantly lower compared to ATS. AT actuation system mass is 2.2% (0.27 × 10^3^ kg) of the total mass (structure + actuators). The maximum force capacity (0.79 × 10^3^ kN) is required for the actuator installed at element 8. The largest length change (−95 mm) is performed by the actuator installed at element 8 under LC1 and LC3. AT has smaller total energy (4.29 × 10^5^ MJ for AT vs 5.80 × 10^5^ MJ for ATS), and it achieves higher savings in mass and energy cost terms (compare Table 11 with Table 10) compared to ATS. Mass and embodied energy savings account for the actuation system share. Therefore, for this configuration, the adaptive tensegrity solution is not as efficient as the equivalent truss system.

### Tensegrity tower configuration

#### Dimensions and boundary conditions

**Fig. 15:**
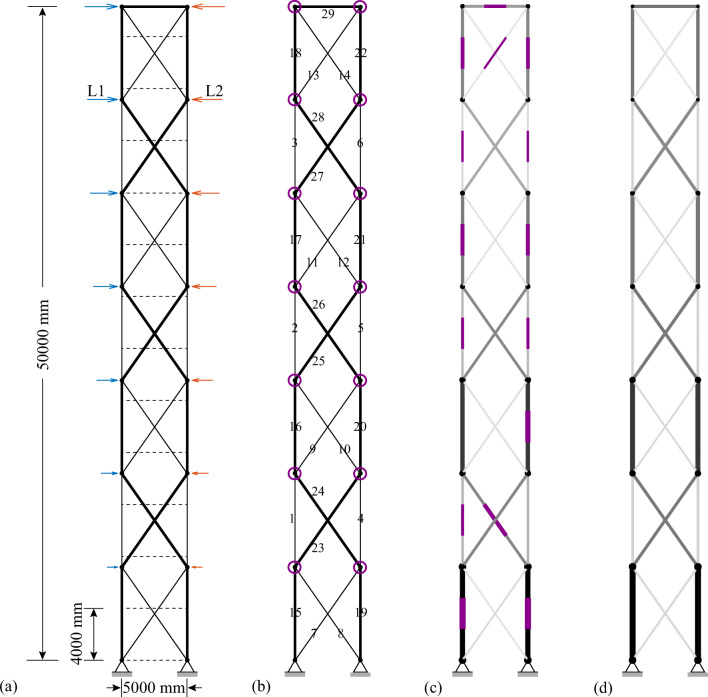
Tensegrity tower: **a** dimensions and loading; **b** element numbering; **c** adaptive tensegrity tower (ATS); and **d** passive tensegrity tower (PTS)

Figure 15a shows the dimensions, support, and loading conditions of the high-rise tensegrity structure considered in this example. The structure has a height of 50 m and a width of 5 m. The dashed lines indicate the story floors. Figure 15b shows the element numbering and the controlled nodes which are indicated by circles. The horizontal displacements of all nodes except the supports are controlled resulting in a total of 14 *cdofs*. The displacement limit for all c*dofs* is set to height/500 = 100 mm. The number of actuators is set to *n*^*act*^ = 14(*cdofs*) + 1(*s*) = 15 (see Section 7.1).

This structure is assumed to be a multistory building reduced to two dimensions. The dead load is set to 2.94 kN/m^2^ (300 kg/m^2^) resulting in a linearly distributed load of 29.4 kN/m applied every 4 m for each floor. Two live loads resulting from wind action are considered. L1 and L2 are horizontally distributed in opposite directions with a magnitude that varies with the square root of the height. The live load maximum magnitude is set to 2.94 kN/m^2^. All loads are appropriately lumped at the nodes through the tributary area of the elements. Table 12 gives all the design loads and combination cases.

**Table 12:** Summary of load cases, tensegrity tower

Load combination case	Load factor	Permanent load	Load factor	Live load
LC1	1.35	self -weight + dead load	1.5	–
LC2	1.35	self -weight + dead load	1.5	L1 = 2.94 kN/m^2^
LC3	1.35	self -weight + dead load	1.5	L2 = 2.94 kN/m^2^

#### Adaptive vs passive tensegrity

**Fig. 16:**
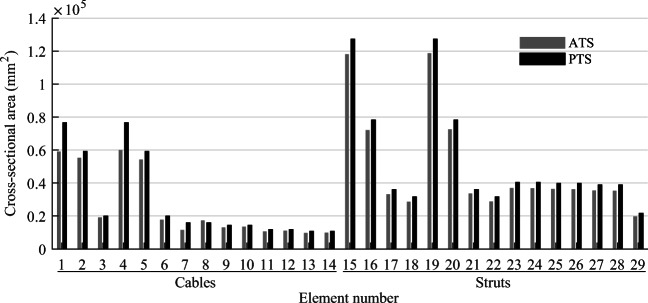
Element cross-sectional areas: adaptive tensegrity tower (ATS) vs passive tensegrity tower (PTS)

Figure 15c and Fig. 15d compare the optimal adaptive tensegrity structure (ATS) obtained through TEO (Section 6) and the passive tensegrity structure (PTS) obtained through the embodied energy minimization method given in Appendix A. Element diameters and cross-sectional areas are indicated by line thickness and color shading variation, respectively. The thicker the line, the bigger the diameter, and a darker gray shade indicates a larger cross-sectional area. The element cross-sectional areas are also indicated by the bar chart in Fig. 16. All ATS elements have a smaller cross-sectional area compared to PTS elements. On average the cross-sectional area of ATS elements is 9.7% smaller than that of PTS elements.

**Fig. 17:**
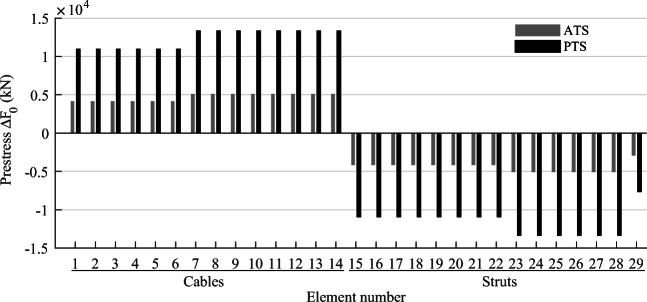
Element prestress: adaptive tensegrity tower (ATS) vs passive tensegrity tower (PTS)

**Fig. 18:**
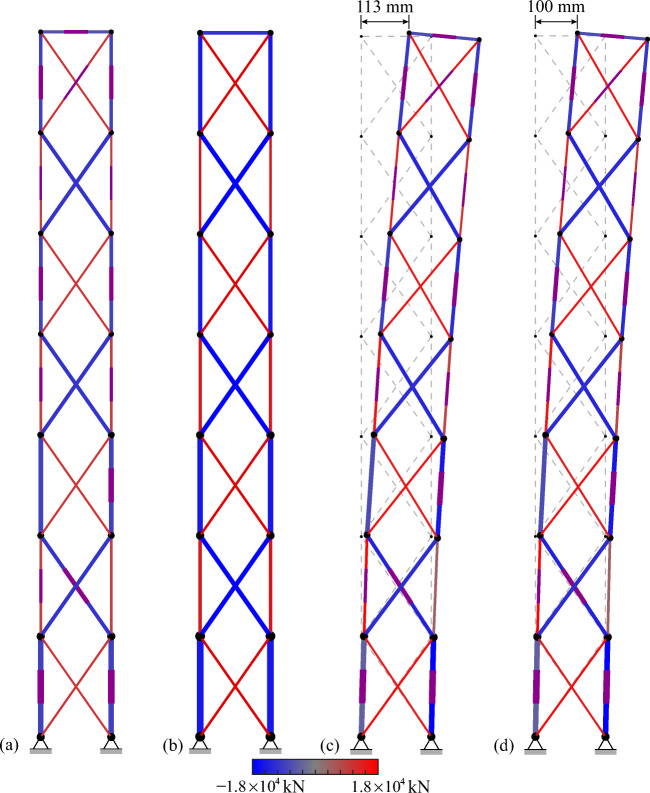
Element prestress for adaptive (**a**) and passive (**b**) tensegrity towers; adaptive tensegrity tower (ATS) deformed (**c**) and controlled (**d**) shape under LC2

Figures 17 and 18a and b show the element prestress through a bar chart and mapped onto the structure geometry, respectively. As expected, for both ATS and PTS, cables are in tension while the struts are in compression, and in both cases, the prestress is proportional to the self-stress state (see Table 8). However, since cable elements can be kept in tension through active control, the prestress required in ATS is 61.9% smaller than that required in PTS.

**Fig. 19:**
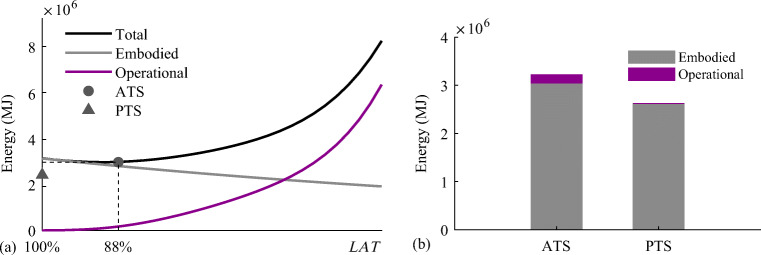
Adaptive tensegrity tower: (**a)** embodied, operational, and total energy vs *LAT*; (**b)** energy comparison optimal adaptive (ATS) vs passive (PTS) solution

Figure 19a shows the plot of embodied, operational, and total energy as functions of *LAT*. PTS and ATS are indicated by a triangle and a circle mark, respectively. ATS is obtained for a *LAT* of 88% which is equivalent to a live load of 2.59 kN/m^2^ (see Table 12). Structural adaptation is required for 3.19 × 10^3^ h under LC2 and LC3 (approximately 4.4 months over a 50-year service life). The *LAT* for PTS is 100% because it is sized so that both ultimate and serviceability limit states are satisfied under the worst load case.

Figure 19b compares adaptive and passive configurations in energy cost terms. ATS requires larger total energy (−22.63%) as well as mass (−16.17%) compared to PTS. Mass and embodied energy savings account for the actuation system share. ATS actuation system mass is 22.2% (1.86 × 10^4^ kg) of the total mass (structure + actuators). The maximum force capacity (−1.3 × 10^4^ kN) is required for the actuator installed at element 24. The largest length change (95 mm) is performed by the actuator installed at element 21 under all load cases. Table 13 gives the optimization metrics for adaptive and passive tensegrity solutions.

**Table 13:** Adaptive tensegrity tower, summary of results

	*LAT*	Mass (kg)	Mass saving	Embodied energy (MJ)	Operational energy (MJ)	Energy saving	Actuation time (hours)	Computation time (seconds)
ATS	88%	8.36 × 10^4^	−16.2%	3.05 × 10^6^	1.70 × 10^5^	−22.6%	3.19 × 10^3^	5273.36
PTS	100%	7.20 × 10^4^	–	2.63 × 10^6^	–	–	–	0.22

**Fig. 20:**
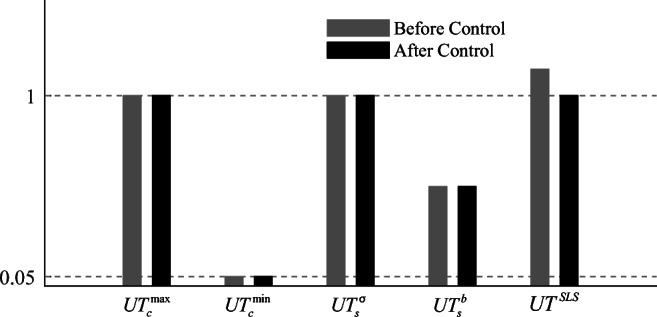
Adaptive tensegrity tower: utilization factors before and after control

Figure 20 shows the utilization factors bar chart (Section 7.2) for ATS before and after control. Cable elements do not slack under any load case; i.e., ${UT}_c^{\mathrm{min}}$ is never smaller than the set lower bound *ζ* = 0.05. Cable and strut forces are within admissible limits (${UT}_c^{\mathrm{max}}$, ${UT}_s^{\sigma }$), and strut forces are lower than the buckling limit (${UT}_s^b$). As expected, *UT*^*SLS*^ reduces to 1.0 through active control. Figure 18c and d show the ATS deformed and controlled shapes, respectively, under load case LC2. Before control, the topmost node displacements exceed deflection limits (100 mm); after control, all node displacements satisfy SLS.

#### Adaptive tensegrity vs equivalent truss system

**Fig. 21:**
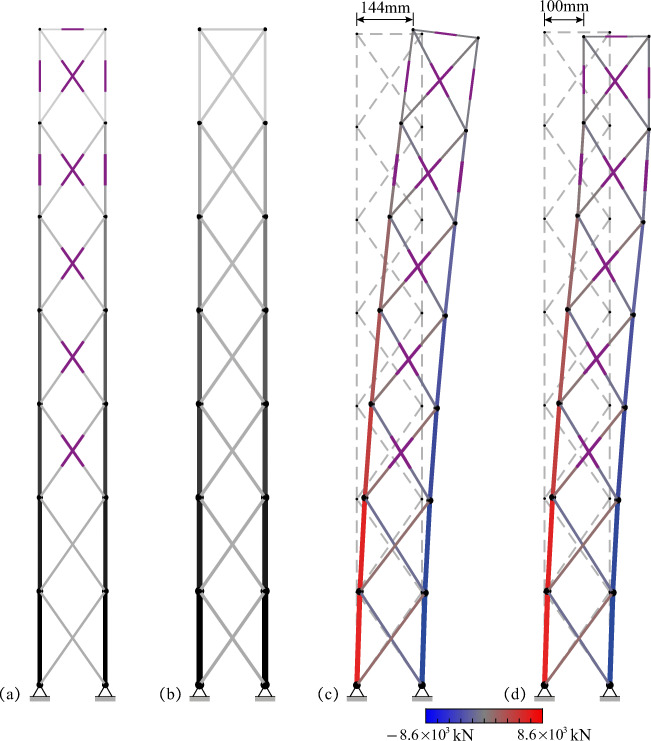
Optimal solutions: (**a**) adaptive truss tower (AT); (**b**) passive truss tower (PT); (**c**) AT deformed; and (**d**) controlled shape under LC2

TEO is applied to design an equivalent truss structure to benchmark mass and energy savings of the adaptive tensegrity solution. Dimensions, element material, topology, loading, and boundary conditions are identical to the tensegrity high-rise structure considered in Section 7.5.1. The optimal passive truss configuration is obtained using the formulation given in Appendix A. The optimal adaptive truss system (AT) and corresponding passive truss system (PT) are shown in Fig. 21. Optimization metrics are given in Table 14.

**Table 14:** Adaptive truss tower, summary of results

	*LAT*	Mass (kg)	Mass saving	Embodied energy (MJ)	Operational energy (MJ)	Energy saving	Actuation time (hours)	Computation time (seconds)
AT	70%	4.01 × 10^4^	29.2%	1.46 × 10^6^	2.39 × 10^5^	17.6%	9.85 × 10^3^	2.73
PT	100%	5.66 × 10^4^	–	2.07 × 10^6^	–	–	–	0.20

AT is obtained for an *LAT* of 70%, which results in a larger actuation time compared to that required by the adaptive tensegrity solution (ATS). However, AT achieves 30% mass savings and 18% energy savings compared to PT. AT actuation system requirements are significantly lower compared to ATS. AT actuation system mass is 2.0% (0.79 × 10^3^ kg) of the total mass (structure + actuators). The maximum force capacity (0.85 × 10^3^ kN) is required for the actuator installed at elements 25 and 26. The largest length change (−19 mm) is performed by the actuator installed at element 18 under LC2. Mass and embodied energy savings account for the actuation system share. In addition, AT has smaller total energy compared to ATS (1.43 × 10^6^ for AT vs 2.37 × 10^6^ for ATS, compare Table 14 with Table 13). Therefore, similar to the roof structure example, the adaptive tensegrity tower is not as efficient as the equivalent truss system in mass and energy cost terms.

### On energy requirements of adaptive tensegrity structures

Further investigation has been carried out for the high-rise structure example to evaluate energy requirements of adaptive tensegrity compared to equivalent truss systems. Figure 21c and Fig. 21d show deformed and controlled shapes, respectively, for AT under LC2. Before control, the top four-node displacements exceed deflection limits, and the deformed shape has a single curvature. After control, all node displacements are reduced within the required limit; the controlled shape, in this case, has a double curvature. Comparing Fig. 21c with d shows that only the shape of the top part of the structure is controlled while the rest of the shape remains nearly unchanged. For AT, the control of the top node displacements does not significantly affect the response of the rest of the truss system, and thus the actuators located in the lower region are not required to apply large forces. Differently for ATS, this control strategy is not applicable. As shown in Fig. 18c and d, deformed and controlled shapes have a single curvature. The tensegrity structure is a closely coupled system, and thus, it is hard to control only selected node displacements without affecting the response in other regions of the structure. It is not possible to control only the displacements of the top nodes because it will cause some of the cables to slack.

**Fig. 22:**
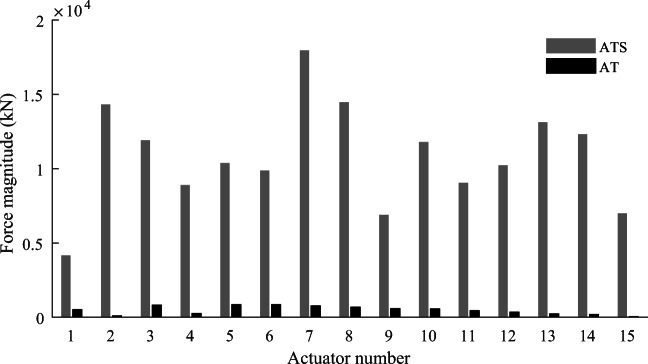
Actuator forces in adaptive tensegrity (ATS) vs adaptive truss (AT)

To avoid large operational energy for control, ATS is obtained for a higher *LAT* (90%) compared to AT (70%) which ultimately results in larger total energy. Figure 22 compares the magnitude of actuator forces for the adaptive tensegrity solution (ATS) and the equivalent truss system (AT). The actuators in ATS have to work against much larger forces compared to the actuators in AT which results in a larger embodied energy for ATS actuation system. Referring to Tables 13 and 14, the actuation system mass and maximum force capacity required in AT (0.79 × 10^3^ kg, 0.85 × 10^3^ kN) are much lower compared to ATS (1.86 × 10^4^ kg, −1.3 × 10^4^ kN). Larger element forces require larger cross-sectional areas, which results in a larger structure embodied energy and mass (compare Table 13 with Table 14). AT structure mass (4.01 × 10^4^ kg) is approximately half compared to ATS (8.36 × 10^4^ kg).

If operational energy is not of primary concern, which could be the case if it is supplied from renewable sources, TEO methodology can be employed to obtain lightweight and low embodied energy configurations. Table 15 compares the optimal adaptive tensegrity solution (*LAT* = 88%) with three other solutions obtained for *LAT* set to 50%, 20%, and 0%. While total energy savings worsen because actuation time and thus operational energy increase significantly, embodied energy and mass savings increase up to 42.59% as *LAT* decreases from the optimal value to *LAT* = 0%. Table 16 compares the optimal adaptive truss solution (*LAT* = 70%) with the other three solutions obtained for *LAT* set to 50%, 20%, and 0%. Similarly, while total energy savings worsen, embodied and mass savings increase up to 73.65% as *LAT* decreases from the optimal value to *LAT* = 0%.

**Table 15:** Adaptive tensegrity tower obtained for different *LAT*s, summary of results

	*LAT*	Mass (kg)	Mass saving	Embodied energy (MJ)	Operational energy (MJ)	Energy saving	Actuation time (h)	Computation time (s)
ATS	0%	4.130 × 10^4^	42.59%	1.507 × 10^6^	3.81 × 10^7^	−1408%	7.22 × 10^4^	978.70
	20%	4.133 × 10^4^	42.55%	1.508 × 10^6^	3.80 × 10^7^	−1404%	7.22 × 10^4^	12,599.25
	50%	5.661 × 10^4^	21.24%	2.066 × 10^6^	4.90 × 10^6^	−186%	2.21 × 10^4^	1183.93
	88%	8.357 × 10^4^	−16.17%	3.050 × 10^6^	1.70 × 10^5^	−22.6%	3.19 × 10^3^	5273.36
PTS	100%	7.194 × 10^4^	–	2.626 × 10^6^	–	–	–	0.21

**Table 16:** Adaptive truss tower obtained for different *LAT*s, summary of results

	*LAT*	Mass (kg)	Mass saving	Embodied energy (MJ)	Operational energy (MJ)	Energy saving	Actuation time (h)	Computation time (s)
AS	0%	1.492 × 10^4^	73.65%	0.544 × 10^6^	1.02 × 10^7^	−419.13%	7.22 × 10^4^	702.12
	20%	1.494 × 10^4^	73.60%	0.545 × 10^6^	9.38 × 10^6^	−380.60%	6.58 × 10^4^	879.96
	50%	2.855 × 10^4^	49.57%	1.042 × 10^6^	1.24 × 10^6^	−10.22%	2.21 × 10^4^	1.57
	70%	4.007 × 10^4^	29.22%	1.463 × 10^6^	2.39 × 10^5^	17.64%	9.85 × 10^3^	2.73
PS	100%	5.661 × 10^4^	–	2.066 × 10^6^	–	–	–	0.20

Note that for all configurations, the truss system (AT) performs significantly better than the tensegrity system (ATS) in mass and energy cost terms, including the passive solution obtained for *LAT* = 100% (compare PTS with PT), which agrees with the findings given in (Nanayakkara et al. 2020).

### On solution quality

Designing adaptive structures through total energy optimization (TEO, Section 6) involves solving embodied energy minimization (Section 4) which is a non-convex mixed-integer nonlinear problem (MINLP) as well as operational energy minimization (Section 5) which is a non-convex nonlinear programming problem (NLP). Embodied and operational energy minimization are nested within an outer optimization process; thus, this methodology cannot guarantee solution optimality. However, it was proven numerically that the all-in-one formulation given in (Wang and Senatore 2020a) and the nested formulation given in (Senatore et al. 2019) produce very similar solutions in energy cost terms. Since the methodology given in this work combines the best aspects of the formulations given in (Senatore et al. 2019; Wang and Senatore 2020a), it is reasonable to assume the same conclusion holds.

Embodied energy minimization for the adaptive configuration is a non-convex MINLP problem that involves a large number of variables and constraints. Therefore, it is generally not possible to obtain a global optimum within a reasonable computation time. An attempt to obtain a global optimum solution for both configurations (tensegrity roof and tower) was made using “BARON” (branch-and-reduce optimization navigator) (Tawarmalani and Sahinidis 2004) which is regarded as one of the best solvers for global optimization of non-convex problems (Mixed Integer Nonlinear Programming Benchmark (MINLPLIB) n.d.). However, no feasible solution was obtained within 8-h computation time. Local optimum solutions have been obtained using other MINLP solvers such as Knitro (Nocedal 2006), Bonmin (Bonami et al. 2008), and FilMINT (Abhishek et al. 2006), which, although cannot guarantee global optimality for non-convex MINLPs, perform well on large-scale problems (Bussieck and Vigerske 2010). Table 17 compares the solutions obtained using different solvers for the roof and tower tensegrity configurations. The computation time limit has been set to 8 h (28,800 s) for all solvers. The *LAT* has been set to 88%, which is the optimal value obtained through TEO for both roof and tensegrity configurations. The dash symbol “-” indicates that no feasible solution could be obtained within the prescribed time limit. The best solutions have been obtained using the branch-and-bound algorithm implemented in Knitro. All simulations have been carried out using the solver default settings.

**Table 17:** Embodied energy minimization (MINLP) solutions by Baron, Knitro, Bonmin, and FilMINT

Embodied energy minimization (MINLP)	Solver	Objective function *E*_*emb*_(MJ)	Computation time (s)
Tensegrity roof	BARON	–	–
	Knitro	6.21 × 10^5^	48.55
	Bonmin	6.21 × 10^5^	2947.49
	FilMINT	7.49 × 10^5^	63.61
Tensegrity tower	BARON	–	–
	Knitro	3.05 × 10^6^	4953.72
	Bonmin	–	–
	FilMINT	–	–

A further benchmark has been carried out to evaluate the quality of solutions produced by Knitro built-in algorithms to solve MINLP problems, which are branch-and-bound (BNB), hybrid Quesada-Grossman (HQG), and mixed-integer sequential quadratic programming (MISQP). Table 18 compares the solutions obtained with these methods. Each algorithm has been tested five times. All simulations have been carried out using the solver default settings. The computation time given in the table is the average value among all tests. Knitro-BNB algorithm produces the best solution within the shortest computation time for both configurations.

**Table 18:** Embodied energy minimization (MINLP) solutions by Knitro MINLP solvers

Embodied energy minimization (MINLP)	Algorithm	Objective function *E*_*emb*_(MJ)	Computation time (s)
Tensegrity roof	Knitro-BNB	6.21 × 10^5^	48.55
	Knitro-HQG	6.26 × 10^5^	113.27
	Knitro-MISQP	–	–
Tensegrity tower	Knitro-BNB	3.05 × 10^6^	4953.72
	Knitro-HQG	–	–
	Knitro-MISQP	–	–

Operational energy minimization for the adaptive configuration and embodied energy minimization for the passive configuration are non-convex NLP problems that have been solved using the interior-point method (IPM) implemented in Knitro. Generally, IPMs cannot guarantee the global optimality of a non-convex NLP problem. To evaluate solution quality, operational energy minimization solutions produced by Knitro-IPM have been benchmarked with solutions obtained through multiple-starting-point search (Knitro-IPM with multi-start option) and NLP-global-solver BARON. All simulations have been carried out using the solver default settings. Solution optimization metrics are given in Table 19. All algorithms have produced an identical solution. The computation time required by Knitro-IPM (multi-start) and BARON is significantly larger compared to Knitro-IPM, thus showing that for this NLP problem, Knitro-IPM is an efficient method to produce high-quality solutions.

**Table 19:** Operational energy minimization (NLP) solutions by Knitro-IPM, Knitro multi-start, and Baron

Operational energy minimization (NLP)	Solver	Objective function *E*_*op*_(MJ)	Computation time (s)
Tensegrity roof	Knitro-IPM	2.83 × 10^4^	0.038
	Knitro-IPM (multi-start)	2.83 × 10^4^	8.25
	BARON	2.83 × 10^4^	0.37
Tensegrity tower	Knitro-IPM	1.70 × 10^5^	0.095
	Knitro-IPM (multi-start)	1.70 × 10^5^	29.13
	BARON	1.70 × 10^5^	35.50

## Discussion

Total energy optimization (TEO) has been formulated to enable the design of minimum energy configurations whether tensegrity or truss systems (Section 6). However, if minimum weight is the primary design objective, TEO can be employed to obtain least-weight solutions by ignoring the operational energy term. Given a fixed number of actuators, minimum weight solutions are obtained for *LAT =* 0% (see Tables 15 and 16). That being said, as the *LAT* decreases from 100 to 0%, the adaptive configuration requires active compensation of displacements under low-intensity and thus more frequent loading events. For this reason, consideration of fatigue becomes important. Future work could look into extending TEO by adding a fatigue limit state to the optimization constraints.

In the numerical examples presented in this paper, the total number of actuators has been predetermined and kept constant during optimization to reduce the solution space size (see Section 7.1). However, the embodied energy minimization formulation (Table 1) allows treating not only the position but also the number of actuators as a variable. Future work could investigate in greater detail the relation between *LAT* and the optimal number of actuators with regard to mass and energy savings.

TEO can be applied to design adaptive tensegrity structures that do not contain mechanisms, i.e., kinematically determinate systems. For practical applications, it might be preferable to avoid kinematic indeterminacy since, generally, it offers no benefits with regard to stiffness and stability of the structure. As remarked by Calladine (Calladine 1978): “On the other hand, if the aim is to design economical but stiff engineering structures it is not clear that there is much point in making the outer network so sparse that the resulting frame has a number of infinitesimal modes whose stiffness is necessarily low.” For example, the deployable pedestrian bridge studied in (Veuve et al. 2015), Georgia Dome (Levy 1994), and Kurilpa Bridge (Beck 2012) are all kinematically determinate tensegrity systems. That being said, for the sake of generality, future work could extend TEO to adaptive kinematically indeterminate systems by incorporating a prestress design process that enables stabilization of first-order infinitesimal mechanisms such as that given in (Wang and Senatore 2020b).

TEO has been formulated with the assumption of small deformations. In this context, the global stability of a kinematically determinate tensegrity structure is ensured through the force unilateral condition on cable elements which are kept in tension under all loading events with and without contribution of the active system. Future work could extend TEO to include geometric nonlinear effects such as large displacements and load direction dependency with deformations.

## Conclusions

This paper has presented a new methodology to design adaptive truss as well as tensegrity structures through total energy optimization (TEO). TEO has been applied to design a roof and a high-rise adaptive tensegrity structure. Benchmark studies have shown that truss systems perform significantly better than tensegrity systems in mass and energy cost terms, whether they are adaptive or passive solutions. Compared to adaptive truss solutions, adaptive tensegrity structures require a larger embodied and operational energy to carry larger forces and to maintain stable equilibrium (i.e., cable elements do not slack and must carry a tension or, at the limit, a zero force). This result is significant, and this is the first paper that has presented it so far. Prior to this work, there was no quantitative study to assess the mass and energy requirements of an adaptive tensegrity structure compared to equivalent passive solutions. Future work could look into applying TEO to other tensegrity configurations to generalize this conclusion.

The effect of structural adaptation has been rigorously investigated by comparing adaptive and passive tensegrity solutions (see Section 7.3). If operational energy is not of primary concern, which could be the case if energy is supplied from renewable resources, TEO produces minimum weight and low embodied energy adaptive tensegrity structures that outperform passive tensegrity structures. This result generalizes conclusions reached in previous work (Senatore et al. 2019; 2018a) to tensegrity systems, thus contributing to extend the domain of application of adaptive structures. The ability to control deflections is particularly beneficial to tensegrity systems which are generally comparatively flexible compared to trusses and frames. In addition, since cable elements can be kept in tension through active control, adaptive tensegrity structures require a much lower prestress (up to 60% lower for the case studies presented in this work) compared to passive tensegrity structures, which reduces construction costs.

Future work could look into extending the TEO formulation to include geometry and topology optimization which might lead to new types of load-bearing adaptive structures and the discovery of as-yet-unknown lower bound configurations.

## Figures and Tables

**Table 20:**
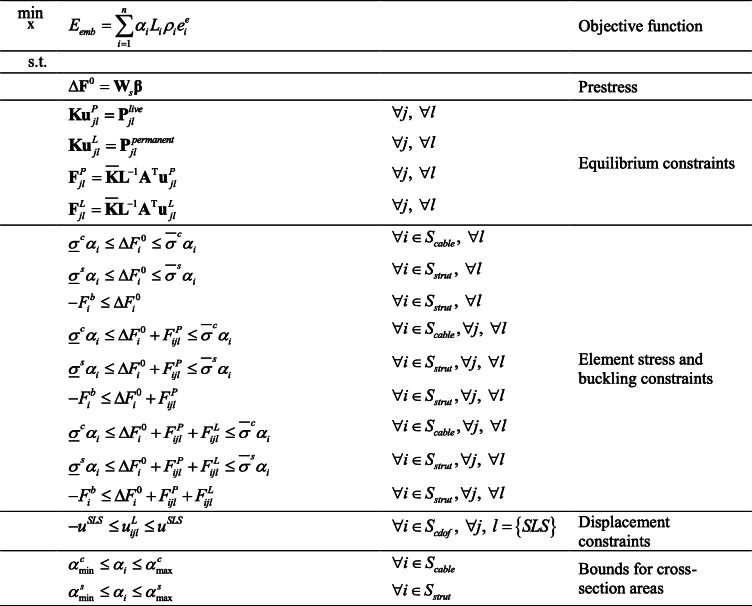
Embodied energy minimization formulation for passive tensegrity and truss structures

## Data Availability

All formulations given in this work have been implemented in Matlab. The optimization problems have been solved through suitable algorithms (see Section 7.7) hosted on the NEOS server (https://www.neos-server.org/neos/). All data including source code is available upon request from the corresponding author. For up-to-date contact information, visit http://www.gennarosenatore.com.

## Appendix

Embodied energy minimization formulation for a passive tensegrity as well as a passive truss configuration is given in Table 20. This formulation applies directly to truss structures by removing the prestress state from the variables and the unilaterality condition on element forces from the constraints. The vector **X** = (**α**, **β**, *Δ***F**^0^, **F**^*P*^, **u**^*P*^, **F**^*L*^, **u**^*L*^) collates all optimization variables. Objective function and all constraint equations have been described in Sections 4 and 5. Unless otherwise indicated, the indices *i*, *j*, and *l* iterate over *n*^*e*^ structural elements, *n*^*p*^ load cases, and the load combination cases {*ULS*, *SLS*}.

### Glossary

$$ \mathbf{A}\in {\mathrm{\mathbb{R}}}^{n^f\times {n}^e} $$: equilibrium matrix
*c*: ratio of actuator mass to actuator force capacity
*cdof*: controlled degree of freedom
$\mathbf{e}\in {\mathrm{\mathbb{R}}}^{n^e}$: element elastic length change
*e*^*a*^: actuator material energy intensity factor
*e*^*e*^: element material energy intensity factor
${\mathbf{e}}^t\in {\mathrm{\mathbb{R}}}^{n^e}$: element total length change
*E*: Young modulus
${E}_{emb}^a$: actuator system embodied energy
${E}_{emb}^s$: structure embodied energy (i.e., without actuators)
*E*_*emb*_: structure total embodied energy (i.e., with actuators)
*E*_*op*_: operational energy
$$ \mathbf{F}\in {\mathrm{\mathbb{R}}}^{n^e} $$: element forces
${\mathbf{F}}^b\in {\mathrm{\mathbb{R}}}^{n^e}$: Euler buckling load
${\mathbf{F}}^L\in {\mathrm{\mathbb{R}}}^{n^e}$: element forces caused by live load
${\mathbf{F}}^{L\_C}\in {\mathrm{\mathbb{R}}}^{n^e}$: controlled element forces under live load **F**^*L* _ *C*^ = **F**^*L*^ + Δ**F**^*L*^
${\mathbf{F}}^P\in {\mathrm{\mathbb{R}}}^{n^e}$: element forces caused by permanent load
${\mathbf{F}}^{P\_C}\in {\mathrm{\mathbb{R}}}^{n^e}$: controlled element forces under permanent load **F**^*P* _ *C*^ = **F**^*P*^ + Δ**F**^*P*^
${\mathbf{F}}^{\psi}\in {\mathrm{\mathbb{R}}}^{n^e}$: element forces in state *ψ*
$\tilde{\mathbf{F}}\in {\mathrm{\mathbb{R}}}^{n^e}$: actuator force capacity
${\tilde{F}}_{\mathrm{min}}$: lower bound for actuator force capacity
${\tilde{F}}_{\mathrm{max}}$: upper bound for actuator force capacity
$\mathbf{G}\in {\mathrm{\mathbb{R}}}^{n^f\times {n}^f}$: element flexibility matrix
$$ \mathbf{K} \in {\mathrm{\mathbb{R}}}^{n^f\times {n}^f} $$: stiffness matrix
*LAT*: load activation threshold
$$ \mathbf{L}\in {\mathrm{\mathbb{R}}}^{n^e} $$: element length
$\mathbf{n}\in {\left\{0,1\right\}}^{n^e\times 1}$: binary variable vector for actuator positions
*n*^*a*^: number of actuators
*n*^*c*^: number of supports
*n*^*d*^: number of samples in the load probability distribution
*n*^*e*^: number of structural elements
*n*^*f*^: number of free degrees of freedom
*n*^*n*^: number of nodes (joints)
*n*^*k*^: number of live load occurrences of intensity larger than *LAT*
*n*^*p*^: number of load cases
$\mathbf{P}\in {\mathrm{\mathbb{R}}}^{n^f}$: total load containing external and actuator load **P** = **P**^*ext*^ + **P**^*act*^
${\mathbf{P}}^{act}\in {\mathrm{\mathbb{R}}}^{n^f}$: equivalent load caused by actuation (actuator load)
$$ {\mathbf{P}}^{ext}\in {\mathrm{\mathbb{R}}}^{n^f} $$: external load
${\mathbf{P}}^L\in {\mathrm{\mathbb{R}}}^{n^f}$: total loading (including actuator load) for live load case
$$ {\mathbf{P}}^{live}\in {\mathrm{\mathbb{R}}}^{n^f} $$: live load
${\mathbf{P}}^P\in {\mathrm{\mathbb{R}}}^{n^f}$: total loading (including actuator load) for permanent load case
$$ {\mathbf{P}}^{permanent}\in {\mathrm{\mathbb{R}}}^{n^f} $$: permanent load
*R*^*e*^: external radius of element cross section
*R*^*i*^: internal radius of element cross section
*s*: number of self-stress states (i.e., degree of static indeterminacy)
*SD*: standard deviation of normal probability distribution
*S*_*act*_: index set for actuator positions (extracted from $\mathbf{n}\in {\left\{0,1\right\}}^{n^e\times 1}$)
*S*_*cable*_: index set for strut elements
*S*_*strut*_: index set for cable elements
**S**_*f*_: force influence matrix
**S**_*u*_: shape influence matrix
*S*_*cdof*_: index set for controlled degrees of freedom
*t*: element cross-section thickness
${\mathbf{u}}^L\in {\mathrm{\mathbb{R}}}^{n^f}$: node displacements caused by live load
${\mathbf{u}}^{L\_C}\in {\mathrm{\mathbb{R}}}^{n^f}$: controlled node displacements under live load **u**^*L_C*^ = **u**^*L*^ + Δ**u**^*L*^
${\mathbf{u}}^P\in {\mathrm{\mathbb{R}}}^{n^f}$: node displacements caused by permanent load
${\mathbf{u}}^{P\_C}\in {\mathrm{\mathbb{R}}}^{n^f}$: controlled node displacements under permanent load **u**^*P_C*^ = **u**^*P*^ + Δ**u**^*P*^
${\mathbf{u}}^{\psi}\in {\mathrm{\mathbb{R}}}^{n^e}$: node displacements in state *ψ*
${\mathbf{u}}^{SLS0}\in {\mathrm{\mathbb{R}}}^{n^f}$: serviceability limit (deflection) under permanent load
${\mathbf{u}}^{SLS}\in {\mathrm{\mathbb{R}}}^{n^f}$: serviceability limit under live load
$\mathbf{W}\in {\mathrm{\mathbb{R}}}^{n^a}$: total work done by actuators
${\mathbf{W}}^F\in {\mathrm{\mathbb{R}}}^{n^a}$: actuator work share under compatible force **F** (before shape control)
${\mathbf{W}}^{\Delta F}\in {\mathrm{\mathbb{R}}}^{n^a}$: actuator work share for force correction Δ**F** (statically indeterminate configurations)
${\mathbf{W}}_s\in {\mathrm{\mathbb{R}}}^{n^e\times s}$: self-stress state matrix.
$\boldsymbol{\upalpha} \in {\mathrm{\mathbb{R}}}^{n^e}$: element cross-section area.
${\alpha}_{\mathrm{min}}^c$: lower bound for cable cross-sectional area.
${\alpha}_{\mathrm{min}}^s$: lower bound for strut cross-sectional area
${\alpha}_{\mathrm{max}}^c$: upper bound for cable cross-sectional area
${\alpha}_{\mathrm{max}}^s$: upper bound for strut cross-sectional area
**β** ∈ ℝ^*s*^: combination factor for self-stress states
*γ*: ratio of cross-section thickness to external radius
$\Delta {\mathbf{F}}^0\in {\mathrm{\mathbb{R}}}^{n^e}$: prestress applied through actuator commands Δ**L**^0^
$\Delta {\mathbf{F}}^L\in {\mathrm{\mathbb{R}}}^{n^e}$: force correction under live load applied through Δ**L**^*L*^
$\Delta {\mathbf{F}}^P\in {\mathrm{\mathbb{R}}}^{n^e}$: force correction under permanent load applied through Δ**L**^*P*^
$\Delta {\mathbf{L}}^0\in {\mathrm{\mathbb{R}}}^{n^e}$: actuator commands to apply prestress Δ**F**^0^
$\Delta \mathbf{L}\in {\mathrm{\mathbb{R}}}^{n^e}$: actuator commands (i.e., control commands)
$\Delta {\mathbf{L}}^P\in {\mathrm{\mathbb{R}}}^{n^e}$: actuator commands under permanent load
$\Delta {\mathbf{L}}^L\in {\mathrm{\mathbb{R}}}^{n^e}$: actuator commands under live load
$\Delta {\mathbf{L}}^{\psi}\in {\mathrm{\mathbb{R}}}^{n^e}$: actuator commands required in in state *ψ*
Δ*L*_limit_: maximum actuator length change
$\Delta {\mathbf{u}}^L\in {\mathrm{\mathbb{R}}}^{n^f}$: displacement correction under live load applied throughΔ**L**^*P*^.
$\Delta {\mathbf{u}}^P\in {\mathrm{\mathbb{R}}}^{n^f}$: displacement correction under permanent load applied throughΔ**L**^*P*^
Δ*t*: duration of loading event in hours
*ζ*: proportional constant for cable admissible stress lower bound
*η*: actuator mechanical efficiency
*μ*: mean of normal probability distribution
*ρ*: material density
${\underline{\sigma}}^c$: cable admissible stress lower bound
${\overline{\sigma}}^c$: cable admissible stress upper bound
${\underline{\sigma}}^s$: strut admissible stress lower bound
${\overline{\sigma}}^s$: strut admissible stress upper bound
*ω*: actuator working frequency

## References

[CR1] Abhishek K, Leyffer S, Linderoth J (2006) FilMINT: An outer-approximation-based solver for nonlinear mixed integer programs, Technical report ANL/MCS-P1374–0906, Argonne National Laboratory, Mathematics and Computer Science Division

[CR2] Adam B, Smith IFC (2008) Active tensegrity: a control framework for an adaptive civil-engineering structure. Comput Struct 86(23–24):2215–2223

[CR3] Ali NBH, Rhode-Barbarigos L, Albi AP, Smith IFC (2010). Design optimization and dynamic analysis of a tensegrity-based footbridge. Eng Struct.

[CR4] Beck HACJ (2012) Kurilpa Bridge, Images Publishing

[CR5] Böhm M, Wagner J, Steffen S, Sobek W, Sawodny O (2019) Homogenizability of element utilization in adaptive structures, In 15th International Conference on Automation Science and Engineering (CASE), Vancouver

[CR6] Bonami P, Biegler L, Conn A, Cornuejols G, Grossmann I, Laird C, Lee J, Lodi A, Margot F, Sawaya N, Wachter A (2008). An algorithmic framework for convex mixed integer nonlinear programs. Discret Optim.

[CR7] Bussieck MR, Vigerske S (2010) MINLP solver software, Wiley Online Library

[CR8] Calladine C (1978). Buckminster Fuller’s “tensegrity” structures and Clerk Maxwell's rules for the construction of stiff frames. Int J Solids Struct.

[CR9] Chen M, Skelton RE (2020). A general approach to minimal mass tensegrity. Compos Struct.

[CR10] Connelly R (2002). “Tensegrity structures: why are they stable?,” in Rigidity theory and applications.

[CR11] ENERPAC, “E328e Industrial Tools - Europe,” (2016). [Online]. Available: https://www.enerpac.com/en-us/downloads. Accessed 21 April 2021

[CR12] Fest E, Shea K, Domer B, Smith IFC (2003). Adjustable tensegrity structures. J Struct Eng.

[CR13] Gilewski WKJAOP (2015). Applications of tensegrity structures in civil engineering. Procedia Eng.

[CR14] Hammond G, Jones C (2008). Embodied energy and carbon in construction materials. Proc Inst Civil Eng Energ.

[CR15] Huber JE, Fleck NA, Ashby MF (1997). The selection of mechanical actuators based on performance indices. Proc Royal Soc A.

[CR16] Kanno Y (2013). Topology optimization of tensegrity structures under compliance constraint: a mixed integer linear programming approach. Optim Eng.

[CR17] Kmet S, Mojdis M (2015). Adaptive cable dome. J Struct Eng.

[CR18] Levy MP (1994) The Georgia dome and beyond: achieving lightweight-longspan structures, In Spatial, Lattice and Tension Structures, ASCE

[CR19] Li Q, Skelton R, Yan J (2011) Integrating mass and control energy optimization for tensegrity structure, In 2011 2nd International Conference on Intelligent Control and Information Processing, Harbin

[CR20] Li S, Xu X, Tu J, Wang Y, Luo Y (2020). Research on a new class of planar Tensegrity trusses consisting of repetitive units. Int J Steel Structures.

[CR21] Masic M, Skelton R (2004) Optimization of class 2 tensegrity towers, In Smart Structures and Materials 2004: Smart Structures and Integrated Systems, San Diego

[CR22] Masic M, Skelton R, de Oliveira M (2005) Integrated structure and control design of modular tensegrities, In Proceedings of the 44th IEEE Conference on Decision and Control, Seville

[CR23] Michell A (1904). The limits of economy of material in frame-structures. The London, Edinburgh, and Dublin Philosophical Magazine and Journal of Science.

[CR24] Mixed Integer Nonlinear Programming Benchmark (MINLPLIB) (2020), [Online]. Available: http://plato.asu.edu/ftp/minlp.html. [Accessed 15 01 2021]

[CR25] Nanayakkara KIU, He L, Fairclough HE, Gilbert M (2020). A simple layout optimization formulation for load-carrying tensegrity structures. Struct Multidiscip Optim.

[CR26] Nocedal J (2006) Knitro: An integrated package for nonlinear optimization, In Large-Scale Nonlinear Optimization, Springer, p. 35–60

[CR27] Pellegrino S (1993). Structural computations with the singular value decomposition of the equilibrium matrix. Int J Solids Struct.

[CR28] Pellegrino S, Calladine C (1986). Matrix analysis of statically and kinematically indeterminate frameworks. Int J Solids Struct.

[CR29] Quagliaroli M, Malerba P, Albertin A, Pollini N (2015). The role of prestress and its optimization in cable domes design. Comput Struct.

[CR30] Raja M, Narayanan S (2007). Active control of tensegrity structures under random excitation. Smart Mater Struct.

[CR31] Raja M, Narayanan S (2009). Simultaneous optimization of structure and control of smart tensegrity structures. J Intell Mater Syst Struct.

[CR32] Reinhorn AM, Soong TT, Lin RC, Riley MA, Wang YP, Aizawa S, Higashino M (1992) Active bracing system: a full-scale implementation of active control, National Center for Earthquake Engineering Research*.* 14 Aug. 1992*.* Buffalo*.* US

[CR33] Reksowardojo AP, Senatore G (2020) A proof of equivalence of two force methods for active structural control. Mech Res Commun 103:103465

[CR34] Reksowardojo AP, Senatore G, Smith IFC (2019) Experimental testing of a small-scale truss beam that adapts to loads through large shape changes, Front Built Environ*,* vol. 5, no. 93

[CR35] Reksowardojo A, Senatore G, Smith IFC (2020). Design of structures that adapt to loads through large shape changes. J Struct Eng.

[CR36] Rodellar J, Mañosa V, Monroy C (2002). An active tendon control scheme for cable-stayed bridges with model uncertainties and seismic excitation. J Struct Control.

[CR37] Senatore G, Reksowardojo AP (2020) Force and shape control strategies for minimum energy adaptive structures, Front Built Environ, vol. 6, no. 105

[CR38] Senatore G, Duffour P, Winslow P (2018). Energy and cost analysis of adaptive structures: case studies. J Struct Eng (ASCE).

[CR39] Senatore G, Duffour P, Winslow P, Wise C (2018). Shape control and whole-life energy assessment of an “infinitely stiff” prototype adaptive structure. Smart Mater Struct.

[CR40] Senatore G, Duffour P, Winslow P (2019). Synthesis of minimum energy adaptive structures. Struct Multidiscip Optim.

[CR41] Skelton RE, Oliveira MCD (2009). Tensegrity systems.

[CR42] Skelton RE, Fraternali F, Carpentieri G, Micheletti A (2014). Minimum mass design of tensegrity bridges with parametric architecture and multiscale complexity. Mech Res Commun.

[CR43] Sobek W (2016). Ultra-lightweight construction. Int J Space Structures.

[CR44] Tawarmalani M, Sahinidis N (2004). Global optimization of mixed-integer nonlinear programs: a theoretical and computational study. Math Program.

[CR45] Teuffel P (2004). “Entwerfen adaptiver Strukturen,” (doctoral dissertation).

[CR46] Tibert G (2002). “Deployable tensegrity structures for space applications,” (doctoral dissertation).

[CR47] Utku S (2018) Theory of adaptive structures: incorporating intelligence into engineered products, Routledge

[CR48] Veuve N, Safaei SD, Smith IFC (2015). Deployment of a tensegrity footbridge. J Struct Eng.

[CR49] Wang Y, Senatore G (2020). Minimum energy adaptive structures–all-in-one problem formulation. Comput Struct.

[CR50] Wang Y, Senatore G (2020). Extended integrated force method for the analysis of prestress-stable statically and kinematically indeterminate structures. Int J Solid Structures.

[CR51] Wang Q, Senatore G, Jansen K, Habraken A, Teuffel P (2020). Design and characterization of variable stiffness structural joints. Mater Des.

[CR52] Wang Q, Senatore G, Jansen K, Habraken A, Teuffel P (2020). Vibration suppression using variable stiffness and damping structural joints. Frontiers in Built Environment.

[CR53] Wang Y, Xu X, Luo Y (2020). Topology design of general tensegrity with rigid bodies. Int J Solids Struct.

[CR54] Wang Y, Xu X, Luo Y (2020d) Topology-finding of tensegrity structures considering global stability condition, J Struct Eng vol. in press, no. DOI: 10.1061/(ASCE)ST.1943-541X.0002843

[CR55] Weidner S, Kelleter C, Sternberg P, Haase W, Geiger F, Burghardt T, Honold C, Wagner J, Böhm M, Bischoff M, Sawodny O, Binz H (2018). The implementation of adaptive elements into an experimental high-rise building. Steel Construction: Design and Research.

[CR56] Xu X, Luo Y (2010). Force finding of tensegrity systems using simulated annealing algorithm. J Struct Eng.

[CR57] Xu X, Wang Y, Luo Y, Hu D (2018). Topology optimization of tensegrity structures considering buckling constraints. J Struct Eng.

[CR58] Zhang J, Makoto O (2015). Tensegrity structures.

